# The Role of Emotion Regulation, Affect, and Sleep in Individuals With Sleep Bruxism and Those Without: Protocol for a Remote Longitudinal Observational Study

**DOI:** 10.2196/41719

**Published:** 2023-08-24

**Authors:** Sylvia D Kreibig, Maia ten Brink, Ashish Mehta, Anat Talmon, Jin-Xiao Zhang, Alan S Brown, Sawyer S Lucas-Griffin, Ariel K Axelrod, Rachel Manber, Gilles J Lavigne, James J Gross

**Affiliations:** 1 Department of Psychology Stanford University Stanford, CA United States; 2 Department of Psychiatry and Behavioral Sciences Stanford University Stanford, CA United States; 3 Faculty of Dentistry Université de Montréal Montreal, QC Canada

**Keywords:** sleep bruxism, emotion regulation, ecological momentary assessment, rhythmic masticatory muscle activity, heart rate variability, wrist actigraphy

## Abstract

**Background:**

Sleep bruxism (SB) is an oral behavior characterized by high levels of repetitive jaw muscle activity during sleep, leading to teeth grinding and clenching, and may develop into a disorder. Despite its prevalence and negative outcomes on oral health and quality of life, there is currently no cure for SB. The etiology of SB remains poorly understood, but recent research suggests a potential role of negative emotions and maladaptive emotion regulation (ER).

**Objective:**

This study’s primary aim investigates whether ER is impaired in individuals with SB, while controlling for affective and sleep disturbances. The secondary aim tests for the presence of cross-sectional and longitudinal mediation pathways in the bidirectional relationships among SB, ER, affect, and sleep.

**Methods:**

The study used a nonrandomized repeated-measures observational design and was conducted remotely. Participants aged 18-49 years underwent a 14-day ambulatory assessment. Data collection was carried out using electronic platforms. We assessed trait and state SB and ER alongside affect and sleep variables. We measured SB using self-reported trait questionnaires, ecological momentary assessment (EMA) for real-time reports of SB behavior, and portable electromyography for multinight assessment of rhythmic masticatory muscle activity. We assessed ER through self-reported trait questionnaires, EMA for real-time reports of ER strategies, and heart rate variability derived from an electrocardiography wireless physiological sensor as an objective physiological measure. Participants’ trait affect and real-time emotional experiences were obtained using self-reported trait questionnaires and EMA. Sleep patterns and quality were evaluated using self-reported trait questionnaires and sleep diaries, as well as actigraphy as a physiological measure. For the primary objective, analyses will test for maladaptive ER in terms of strategy use frequency and effectiveness as a function of SB using targeted contrasts in the general linear model. Control analyses will be conducted to examine the persistence of the SB-ER relationship after adjusting for affective and sleep measures, as well as demographic variables. For the secondary objective, cross-sectional and longitudinal mediation analyses will test various competing models of directional effects among self-reported and physiological measures of SB, ER, affect, and sleep.

**Results:**

This research received funding in April 2017. Data collection took place from August 2020 to March 2022. In all, 237 participants were eligible and completed the study. Data analysis has not yet started.

**Conclusions:**

We hope that the effort to thoroughly measure SB and ER using gold standard methods and cutting-edge technology will advance the knowledge of SB. The findings of this study may contribute to a better understanding of the relationship among SB, ER, affect, and sleep disturbances. By identifying the role of ER in SB, the results may pave the way for the development of targeted interventions for SB management to alleviate the pain and distress of those affected.

**International Registered Report Identifier (IRRID):**

DERR1-10.2196/41719

## Introduction

### Background

Sleep bruxism (SB) is an oral behavior [1] characterized by high levels of repetitive jaw muscle activity during sleep, known as rhythmic masticatory muscle activity (RMMA), and expressed as teeth grinding and clenching [2]. When its occurrence is frequent and associated with oral damage, pain, or sleep or neurological comorbidities, it can be classified as a sleep-related movement disorder. With a general population prevalence of 5.5% to 16% [3,4], SB varies on a spectrum of frequency, severity, and awareness, with many experiencing subclinical levels of teeth grinding [5]. Despite its detrimental outcomes, including jaw pain and fractured teeth as well as impaired sleep, function, and quality of life, there is currently no cure for SB [6].

SB can be measured in terms of self-reported or physiological measures at either trait or state timescales. Self-reported individual-difference questionnaires ask about perceived symptoms over weeks or months to assess trait-level SB [7]. Momentary questionnaires probe current pain or teeth grinding during the preceding sleep period to assess state-level SB [8]. Although relatively inexpensive, self-reporting sleep behavior limits the validity of this method of assessing SB because individuals are not aware of the behavior unless told by an observer. Physiological measures have the advantage of unobtrusively assessing SB while the individual is asleep. In-laboratory polysomnography is considered the gold standard of objective sleep measurement [9], but it is not feasible for multiple nights. Alternatively, portable physiological devices are optimal for a multinight assessment within a participant’s home because they are relatively inexpensive to purchase, easy to distribute, and ideal for self-application [10,11].

### SB and Emotion Regulation

The etiology of SB is not yet well understood. It is widely thought to have multiple predisposing factors [6], including maladaptive personality traits, neurotransmitter imbalance, and psychosocial impairments. Recent research implicates negative emotions as a likely factor. Tensing of the jaw muscles can be part of an emotional motor response [12], and acute wake-time negative emotions can increase RMMA [13]. SB has also been associated with impaired affect regulation, either diminished use of adaptive regulation strategies or increased use of maladaptive regulation strategies [14-17]. However, this relationship remains unclear [18].

Emotion regulation (ER) is defined as “attempts to influence which emotions we have, when we have them, and how those emotions are experienced and expressed” [19]. Individuals can regulate their emotions by implementing ER strategies [19] such as cognitive change (generating alternative interpretations of an event to change its affective impact [eg, cognitive reappraisal]), attentional deployment (directing one’s attention to other aspects of the situation [eg, distraction]), and response modulation (controlling experiential, behavioral, or physiological emotional reactions [eg, expressive suppression]). Cognitive change and attentional deployment are considered generally adaptive strategies, whereas expressive suppression is more so a generally maladaptive or harmful strategy [20]. One core aspect of ER is how frequently individuals use a specific ER strategy (ie, ER frequency or use). Another core aspect of ER is how individuals perceive their ability to successfully use an ER strategy to achieve the goal of the ER attempt (ie, ER self-efficacy or effectiveness) [21].

Similar to SB, ER can be measured in terms of self-reported or physiological measures at either trait or state timescales. Self-reported trait questionnaires and momentary assessments of ER strategy use tap into the domain of deliberate, reflective, and consciously accessible information, whereas physiological measures provide access to relatively unconscious and automatic processes [22] and circumvent the numerous limitations of self-report. Such limitations include response biases, limited insight, and one’s motivational level. Research has shown a positive association between ER effectiveness and both resting and 24-hour heart rate variability (HRV) [23,24].

### SB and Affective and Sleep Disturbances

Although affective and sleep disturbances play a prominent role in SB, there have been mixed patterns of findings in the current literature. In the domain of affective disturbances, depression, anxiety, negative affect, and perceived stress are key variables that have been related to self-reported, clinician-diagnosed, or physiologically based SB. Depression has been associated with self-reported [25] but not polysomnography-based measurements of SB [26]. Trait anxiety has been linked with self-reported [27] and physiological [28] measures of SB. Negative affect has been found to be greater in individuals with clinician-diagnosed SB than in those without in 1 study [29] but not in another study [25]. Perceived stress has been associated with self-reported or physiological measures of SB in some studies [16,30] but not in others [31,32].

In the sleep domain, sleep quality and sleep efficiency represent key variables that have been related to SB. Self-reported sleep quality, hereinafter referred to as sleep quality, integrates perceived experiences reflecting sleep latency, sleep duration, habitual sleep efficiency, sleep disturbances, the use of sleeping medication, and daytime dysfunction [33]. Some studies have found worse trait sleep quality with self-reported [34] or physiological measures (including polysomnography) [35] of SB, but this was not the case in another multimeasure study [32]. Worse state sleep efficiency (the ratio of time spent in bed asleep to the total amount of time spent in bed) has been found for both self-reported or clinically diagnosed [34] and home-based electromyography (EMG) [36] SB measures but not in a polysomnography sleep laboratory study [37].

The mixed patterns of results of SB in relation to affective and sleep disturbances suggest that an underlying cause may exist. We propose that ER may play this role. In fact, ER is a transdiagnostic factor in affective and sleep disorders [38]. This study proposes to phenotype ER in SB with both self-reported and physiological trait and state measures of SB and ER collected longitudinally in the context of a 14-day ambulatory assessment while controlling for affect and sleep.

### Hypotheses and Objectives

We hypothesize that the effect of ER in those with SB remains when adjusting for associations with affect and sleep variables. Therefore, we expect that for those with greater (vs lower) self-reported or physiological trait or state SB, there will be a lower self-reported trait and state use of cognitive reappraisal and distraction (hypothesis 1a [H1a]), a greater self-reported trait and state use of expressive suppression (H1b), a lower self-reported trait and state effectiveness of cognitive reappraisal and distraction (H2a), a greater self-reported trait and state effectiveness of expressive suppression (H2b), and a lower physiological trait and state HRV (H2c).

The primary objective of this study is to test whether ER is associated with SB, while controlling for affect and sleep variables. As outlined in *Methods* section, we operationalized the core variables of SB, ER, affect, and sleep in terms of self-reported and physiological assessment modalities and trait and state temporal modalities. The secondary objective of the study is to test cross-sectional and longitudinal mediation pathways in the bidirectional relationships among SB, ER, affect, and sleep.

## Methods

### Study Design

**Figure 1:**
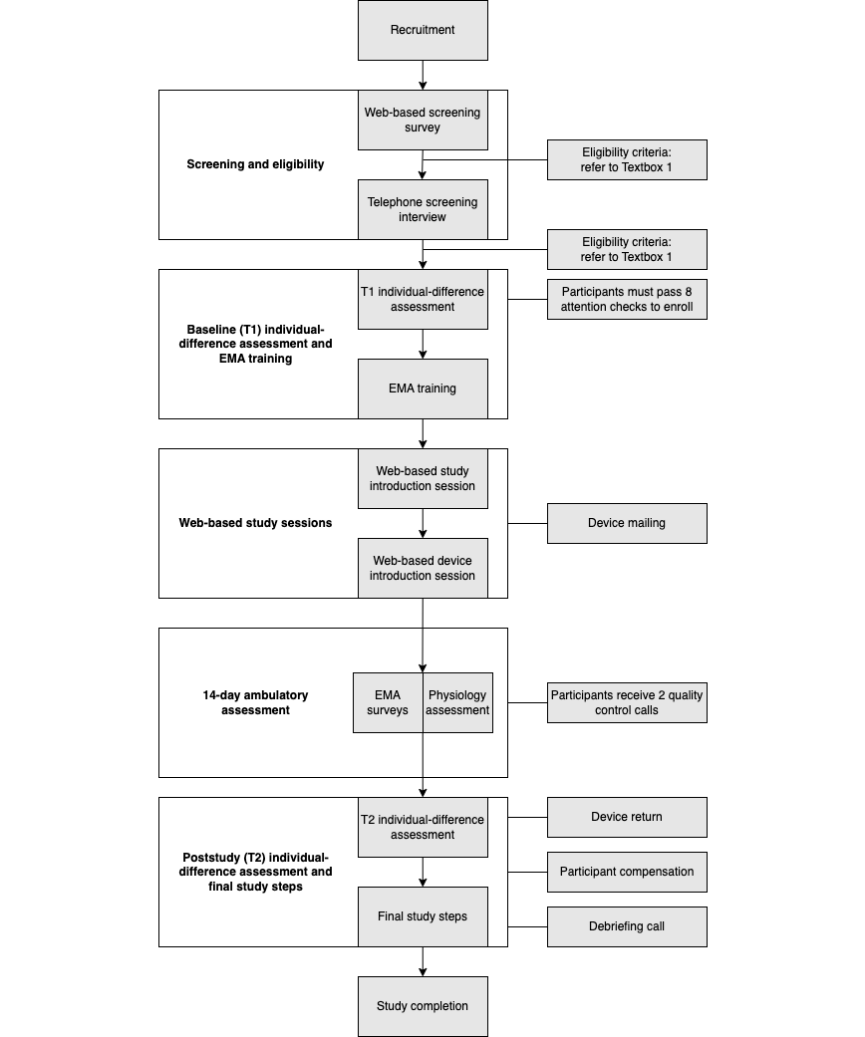
Study design. As indicated in the white boxes, participants underwent 5 task components; criteria and additional procedures are noted to the right of these white boxes. EMA: ecological momentary assessment.

This study applied a repeated-measures observational design. It did not use randomization, blinding, or assignment. As Figure 1 shows, participants went through a fully remote 5-step procedure, including screening and determination of eligibility, an assessment of individual differences, 2 web-based study sessions, the 14-day ambulatory assessment, and an abbreviated assessment of individual differences. As defined by the National Institutes of Health (NIH), this study is a basic behavioral science study at stage 0. It was preregistered at the National Institute of Dental and Craniofacial Research [39] of the NIH on July 8, 2020 (1R01DE026771-01, version 0.1).

### Ethics Approval

The protocol of this study was submitted to, and approved by, Institutional Review Board 7 of Stanford University, Stanford, California, United States (FWA00000935; protocol #50342).

### Informed Consent and Risk Mitigation

Participants were informed of, and consented to, the study’s potential risks, although the overall evaluation of risk was low owing to the use of noninvasive procedures and precautionary measures such as (1) measures taken to preserve participant confidentiality (eg, anonymous participant IDs, a password-protected database, separation of data and identifiable information, and access restrictions to data sets), (2) measures to mitigate risk of suicidal ideation (eg, the use of scripts assessing the severity of suicidality and following referral plans in the case of detected suicidal ideation), (3) measures to mitigate the risk of uncovering an unknown health condition (eg, through the consent form stating that study team members are not responsible for communicating medical diagnoses to participants), (4) measures to mitigate experiencing minor skin irritation from electrodes (eg, the use of clinical-grade gels and adhesives, instructing participants to discontinue use in the case of a reaction, creating an explicit electrode removal and reapplication schedule, sending reminder emails and conducting check-in calls, and requesting participants to contact the study team at the first sign of potential skin irritation), and (5) measures mitigating the risk of negatively affecting participants’ psychological well-being (eg, including a consent statement that explains risks and informs participants of their right to voluntarily withdraw from the study at any time).

### Recruitment and Participants

A minimum sample size of 236 participants was determined by power analyses (G*Power; Heinrich Heine University Düsseldorf) [40]. The calculations were based on the detection of typical effect size estimates of prior studies investigating affect regulation in SB (Cohen *d* >0.21) [14-18], with at least 80% power for 2-tailed pairwise comparisons at Cronbach α ≤.05.

Enrollment and data collection took place from August 2020 to March 2022. Participants were recruited through Facebook advertisement campaigns, research registries, web-based forums, web-based classroom visits, university email mailing lists, and flyers posted at medical clinics and on university campuses on the West Coast. We sought to enroll 275 participants approximately evenly distributed across SB severity and sex, as well as racial and ethnic diversity representative of that in the United States. Racial and ethnic details were collected via the web-based screening survey (Multimedia Appendix 1) that was used to determine study eligibility. We completed intermittent distribution checks to assess the need for targeted recruitment. Participants who completed all steps of the study were compensated with a US $80 gift card as well as a comprehensive sleep report.

### Inclusion and Exclusion Criteria

The inclusion and exclusion criteria are presented in Textbox 1.

**Inclusion and exclusion criteria.**

**Inclusion criteria**

- Living in the United States
- Aged between 18 and 49 years
- High school graduate
- At least 5 natural teeth per quadrant
- Normative bedtime (ie, bedtime no later than 1 AM and wake time no later than 10 AM)
- Refrain from consuming alcohol as well as nicotine and cannabis
- Refrain from consuming caffeine after 12 PM
- Maintain a stable bedtime routine (see 5th point in inclusion criteria)
- Pass at least 8 of 9 attention checks in the 90-minute baseline individual-difference assessment
- Have reliable access to the internet

**Exclusion criteria**

- Reporting as having health conditions other than sleep bruxism (SB), especially physical, mental, and sleep disorders known to affect SB or sleep
- A history of skin allergies
- Any traveling during the 14-day ambulatory assessment that affects circadian rhythm and thus bedtime routine
- Undergoing SB treatment
- Intake of any of the disqualifying medications (ie, antidepressants, anxiolytics, antipsychotics, antihypertensives, thyroid medications, antiasthmatics, anti–Parkinson disease medications, anticonvulsants, antihistamines, triptans, stimulants, and sleep medicines)
- Significant exposure to nicotine or cannabis products
- Caffeine use disorder
- Current pregnancy or pregnancy within the past year or the presence of infants aged <12 months
- Stomach sleeping or uncommon sleeping arrangement that would prevent the application of sensors

### Screening and Eligibility

#### Web-Based Screening Survey

Potential participants were directed through a link from the recruitment medium to the 30-minute web-based screening survey.

#### Telephone Screening Interview

Potential participants who met the inclusion criteria assessed at the web-based screening survey completed a telephone interview to screen for psychiatric (Mini International Neuropsychiatric Interview) [41] and sleep disorders (Duke Structured Interview for Sleep Disorders) [42].

### Materials

Study data were collected and managed using the electronic data capture tool REDCap (Research Electronic Data Capture; Vanderbilt University) hosted at Stanford University [43]. The ecological momentary assessment (EMA) training was presented, and the EMA surveys were collected, using the Qualtrics survey software and platform (versions 09-2020 to 04-2022; Qualtrics). The EMA surveys were delivered using a web application built by our research team (Multimedia Appendix 2). The study sessions were conducted via laptop computers running the secure Zoom software for videoconferencing (version 5.9.1, package 3506; Zoom Video Communications, Inc).

### Baseline Individual-Difference Assessment and EMA Training

#### Baseline Individual-Difference Assessment

After the screening process and before enrolling in the study, participants completed the 90-minute baseline (T1) individual-difference assessment.

#### EMA Training

Upon completion of the T1 individual-difference assessment, participants were directed to the 30-minute EMA training via a web-based link.

### Web-Based Study Sessions and Device Mailing

#### Web-Based Study Introduction Session

In the 30-minute study introduction session, participants were guided through signing the consent form to enroll in the study. Research assistants identified a timetable with the participant with SMS text messaging for the ambulatory assessment, considering the participant’s wake, sleep, and general life schedule.

#### Device Mailing

After the study introduction session, the research team mailed the study equipment to the participants. The package included (1) an RMMA device with 18 pregelled adhesive pads (Butler GrindCare; Sunstar Suisse SA) and 14 alcohol swabs; (2) an electrocardiography (ECG) device (Vivalink VS-US4 - ECG Multi-Vital-Monitor; Vivalink, Inc) with 8 ECG device adhesives (Vivalink Hydrogel Adhesive [VSA-1, A13]; Vivalink, Inc), 10 skin-barrier film wipes (SNS80775; Safe n Simple), and 10 alcohol prep pads (CUR45585RBZ; Curad); and (3) an actigraphy wristwatch (Respironics Actiwatch Spectrum Plus; Philips), configured for the participant’s data collection using the Actiware software (Philips), an instructional brochure, and a return envelope that participants were to use to return the devices to the study team. Each physiology device was fully charged and sanitized before we mailed it out. The study team kept a mailing protocol and recorded the serial numbers of all equipment sent out to participants on a device tracking form.

#### Web-Based Device Introduction Session

The 45-minute device introduction session took place after the participant had received the physiology devices. A research assistant instructed participants on device application, charging, and troubleshooting. The ambulatory assessment began at the end of this session and ended 14 days (336 hours) later.

### 14-Day Ambulatory Assessment and Quality Assurance

#### EMA Surveys

During the 14-day ambulatory assessment, 5 daily EMA surveys were sent to participants: 1 morning diary, 1 evening diary, and 3 daytime surveys (Multimedia Appendix 3 [44,45]). The EMA surveys were delivered using a web application built by our research team. The web application allows research assistants to enroll new participants and create a schedule for when EMA SMS text message reminders with the survey link are delivered to them. This system was hosted on a server (Amazon Web Services, Inc) that provides an on-demand cloud computing platform and application programming interfaces. EMAs were delivered through an automated SMS text messaging system (Twilio). The web application sent an application programming interface request to Twilio at the scheduled times, and Twilio sent predefined EMA SMS text messages to participants. Participants were instructed to complete the morning survey 15 minutes after getting out of bed, the evening survey 30 to 40 minutes before going to bed, and the 3 daytime surveys as soon as possible after they received the SMS text messages. The 3 daytime surveys were spaced at least 3 hours apart. All 5 surveys were set to arrive within a 30-minute window (jittered ±15 minutes from the scheduled time) to prevent the timing of the assessment from being a confounding variable. Participants were given a maximum of 3 hours to complete the surveys and received a follow-up SMS text message after approximately 30 minutes if no response was received. If there were no responses to the EMA surveys for >24 hours, the research team contacted the participant to ensure that there were no technical issues keeping them from completing the EMA surveys.

#### Physiology Assessment

Concurrently with the EMA surveys, participants wore 3 physiological devices for 14 consecutive days and nights (Multimedia Appendix 4). Participants were instructed to apply a facial EMG device each evening at bedtime and remove and charge it each morning after waking; continuously wear an ECG device, except for an approximately 4-hour charging period every 3 days; and continuously wear an actigraphy device. The EMG device was referred to as *Sleep Buddy* in communications with participants to avoid affecting RMMA behavior through expectations.

#### Quality Assurance

Research assistants followed a quality assurance protocol to monitor participant safety and data completeness during the ambulatory assessment, which involved participants receiving 2 quality control calls during the 14-day ambulatory assessment (Figure 1).

### Poststudy Individual-Difference Assessment and Final Study Steps

#### Poststudy Individual-Difference Assessment

The day after their ambulatory assessment ended, participants completed an abbreviated poststudy (T2) individual-difference assessment with questionnaires about affect and sleep.

#### Device Return

At the end of the study, participants were instructed to mail back the physiology devices to the study team with the provided return envelope (Multimedia Appendix 5). Upon receipt, a research assistant exported and saved the data from the devices and remote servers.

#### Participant Compensation

Once data completeness status had been established, a research assistant issued participant compensation (Multimedia Appendix 5).

#### Debriefing Call

Research assistants administered a debriefing call to participants at the end of the study.

### Monitoring of Data Collection

#### Stopping Guidelines

We stopped data collection upon reaching one of the stopping guidelines: (1) 275 participants had been enrolled in the study or (2) the enrollment window of April 1, 2022, had passed.

#### Premature Termination or Suspension of Study

Participants were able to stop participating at any point. The participation of a participant in the study could be terminated by the principal investigator, the institutional review board, or the funder in case of unexpected, significant, or unacceptable risk to participants [46]; insufficient adherence to protocol requirements; or insufficiently complete and evaluable data.

### Measures

We present the core outcome measures among sleep- and affect-related constructs in Tables 1 and 2. At a minimum, participants had to complete the T1 individual-difference assessment, respond to 2 of the 5 EMA surveys each day on 7 of the 14 days, and wear the actigraphy device for 7 of the 14 days.

**Table 1:** Operationalization of sleep-related core constructs through dependent variables.

Core construct, assessment modality, temporal modality, assessment time point, and time frame						Measurement tool
**Sleep bruxism**						
	**Self-report**					
		**Trait**				
			**Baseline (T1) individual-difference assessment**			
				Past 6 months	Sleep Bruxism Questionnaire	
				Past 7 days	PROMIS^a^ Pain Intensity-Short Form 3a v1.0	
				Last year	Oral Health Impact Profile 14	
		**State^b^**				
			**Morning diary survey**			
				Past sleep	Single EMA^c^ item on teeth grinding or clenching	
				Morning (current)	Single EMA item on jaw pain or soreness	
			**Evening diary survey**			
				Past day	Four EMA items adapted from Oral Behavior Checklist on frequency of teeth contact, jaw muscle tensing, teeth clenching, and tongue thrusting	
	**Physiology**					
		**Trait**				
			**Ambulatory assessment**			
				Multinight	Butler GrindCare device (Sunstar Suisse SA) for RMMA^d^ of anterior frontalis muscle bursts	
		**State^b^**				
			**Ambulatory assessment**			
				Nightly	Butler GrindCare device for RMMA of anterior frontalis muscle bursts	
**Sleep**						
	**Self-report**					
		**Trait**				
			**Baseline (T1) and poststudy (T2) individual-difference assessment**			
				Past month	Pittsburgh Sleep Quality Index, sleep quality scale; and Pittsburgh Sleep Quality Index, sleep efficiency scale	
		**State^b^**				
			**Morning diary survey**			
				Past sleep	The Expanded Consensus Sleep Diary for Evening, morning portion on sleep quality; and The Expanded Consensus Sleep Diary for Evening, morning portion on sleep efficiency	
	**Physiology**					
		**Trait**				
			**Ambulatory assessment**			
				Multinight	Respironics Actiwatch Spectrum Plus (Philips) and Actiware software (Philips) for sleep efficiency	
		**State^b^**				
			**Ambulatory assessment**			
				Nightly	Respironics Actiwatch Spectrum Plus and Actiware software for sleep efficiency	

^a^PROMIS: Patient-Reported Outcomes Measurement Information System.

^b^Repeated daily, momentary, and continuous state measures may be averaged over multiple items and assessment time points to form trait measures.

^c^EMA: ecological momentary assessment.

^d^RMMA: rhythmic masticatory muscle activity.

**Table 2:** Operationalization of affect-related core constructs through dependent variables.

Core construct, assessment modality, temporal modality, assessment time point, and time frame						Measurement tool
**Emotion regulation**						
	**Self-report**					
		**Trait**				
			**Baseline (T1) individual-difference assessment**			
				Lifetime	Emotion Regulation Questionnaire, frequency of cognitive reappraisal use subscale; Emotion Regulation Questionnaire, frequency of expressive suppression use subscale; frequency of distraction use; Emotion Regulation Questionnaire, self-efficacy of cognitive reappraisal effectiveness; Emotion Regulation Questionnaire, self-efficacy of suppression; and self-efficacy of distraction	
		**State^a^**				
			**Morning diary survey**			
				Presleep	Single EMA^b^ item on emotion regulation use and single EMA item on emotion regulation effectiveness	
			**Daytime survey**			
				Past 3 hours	Single EMA item on cognitive reappraisal use, single EMA item on expressive suppression use, single EMA item on distraction use, and single EMA item on emotion regulation effectiveness	
	**Physiology**					
		**Trait**				
			**Ambulatory assessment**			
				Multiday	Vital Scout ECG^c^ device (Vivalink) and Kubios HRV Premium software (Kubios Oy) for HRV^d^ (emotion regulation effectiveness)	
		**State^a^**				
			**Ambulatory assessment**			
				24 hours	Vital Scout ECG device and Kubios HRV Premium software for HRV (emotion regulation effectiveness)	
				Resting state	Vital Scout ECG device and Kubios HRV Premium software for HRV (emotion regulation effectiveness)	
**Affect**						
	**Self-report**					
		**Trait**				
			**Baseline (T1) and poststudy (T2) individual-difference assessment**			
				Past 7 days	PROMIS^e^ Short Form v1.0-Depression 8a and PROMIS Short Form v1.0-Anxiety 8a	
				Past 14 or 7 days (T1 and T2)	Positive and Negative Affect Schedule, positive affect subscale; and Positive and Negative Affect Schedule, negative affect subscale	
				Past month	Perceived Stress Scale	
		**State^a^**				
			**Morning diary survey**			
				Current	Single EMA item on positive affect and single EMA item on negative affect	
			**Evening diary survey**			
				Current	Single EMA item on positive affect and single EMA item on negative affect	
				Past day	Single EMA item on positive affect, single EMA item on negative affect, and single EMA item on perceived stress	
			**Daytime survey**			
				Current	Single EMA item on positive affect and single EMA item on negative affect	

^a^Repeated daily, momentary, and continuous state measures may be averaged over multiple items and assessment time points to form trait measures.

^b^EMA: ecological momentary assessment.

^c^ECG: electrocardiography.

^d^HRV: heart rate variability.

^e^PROMIS: Patient-Reported Outcomes Measurement Information System.

### Demographics

We collected information on self-reported sex, age, ethnicity, race, and marital and socioeconomic status in the T1 individual-difference assessment.

### SB Measures

#### Individual-Difference Assessment

We measured self-reported trait SB as follows (Multimedia Appendix 6 [33,47-53]).

The Sleep Bruxism Questionnaire, which was created by our research team based on prior questionnaires [54-56], assesses the perceived experience of SB.

The Patient-Reported Outcomes Measurement Information System (PROMIS) Pain Intensity-Short Form 3a v1.0 [47] measures subjective pain in the past 7 days and at present. It has a Cronbach α value of .906 and demonstrates high construct validity [57,58]. This questionnaire was adapted to ask about (1) orofacial pain and (2) other pain throughout the body.

The Oral Health Impact Profile-14 [48] is a short-form version that measures self-reported dysfunction and distress stemming from one’s oral condition. It has a Cronbach α value of .880 and has strong construct validity [48].

#### Morning and Evening Diary Surveys

We measured self-reported state SB through morning diary surveys composed of single-item questions about past-night teeth grinding and current jaw pain in combination with evening diary surveys composed of 4 questions about teeth behaviors throughout the day that were adapted from the Oral Behaviors Checklist [44] (Multimedia Appendix 3).

#### Ambulatory Assessment

We measured physiological trait and state SB in terms of RMMA using Butler GrindCare (Sunstar Suisse SA), a portable single-channel EMG device (Multimedia Appendix 4). Past research supports the device’s reliability and validity for assessing SB, consistent with gold standard measurement of SB through polysomnography [10,11,59].

### ER Measures

#### Individual-Difference Assessment

We measured self-reported trait ER use and effectiveness as follows (Multimedia Appendix 6).

Two versions of the Emotion Regulation Questionnaire were used. The original version [49] assesses habitual use or frequency of ER strategies in terms of cognitive reappraisal (Cronbach α >.880) and expressive suppression (Cronbach α >.750) with strong overall validity [60]. The additional version assesses habitual use of distraction.

A derived measure [49] assesses the perceived self-efficacy of ER strategy use via reappraisal and suppression subscales (Cronbach α >.870) [61]. The other version assesses self-efficacy of distraction use.

#### Morning Diary and Daytime Surveys

We measured self-reported state ER use and effectiveness through the morning diary survey, which contained questions pertaining to presleep ER. Using the daytime survey, we measured the response to a negative event that had occurred since the last survey (within the past 3 hours) in terms of cognitive reappraisal, distraction, and expressive suppression (Multimedia Appendix 3).

#### Ambulatory Assessment

We measured physiological trait and state ER effectiveness using continuous ECG from a medical-grade wearable and reusable ECG sensor: VS-US4-ECG (Vivalink Inc) (Multimedia Appendix 4). This device has been demonstrated to be reliable and valid [62].

### Affect Measures

#### Individual-Difference Assessment

We measured self-reported trait affect as follows (Multimedia Appendix 6 and 7).

The PROMIS Short Form v1.0-Depression 8a [50] assesses depression in terms of emotional distress caused by depressed mood in the last 7 days. The measure’s Cronbach α values range from .920 to .980 [58], and it demonstrates strong validity [57].

The PROMIS Short Form v1.0-Anxiety 8a [50] assesses anxiety in terms of fear, anxious misery, hyperarousal, and somatic symptoms related to arousal in the last 7 days (Cronbach α=.950) [50], and the tool has strong validity [57].

The Positive and Negative Affect Schedule [51] assesses positive and negative affect over the past 2 weeks via 2 scales. The measure’s Cronbach α values range from .860 to .900 and .840 to .870 for positive and negative affect scales, respectively; it has good validity [51].

The Perceived Stress Scale [52] assesses perceived stress in terms of appraisal of stress in daily life in the past month, where Cronbach α >.700 was reported with adequate validity [63].

#### Morning and Evening Diary and Daytime Surveys

We measured self-reported state affect through single-item questions about current positive and negative affect. We also administered single-item questions about past-day positive and negative affect and perceived stress (evening diary) (Multimedia Appendix 3).

### Sleep Measures

#### Individual-Difference Assessment

We measured self-reported trait sleep quality and efficiency with the Pittsburgh Sleep Quality Index [33], which is composed of seven subscales: (1) subjective sleep quality, (2) sleep latency, (3) sleep duration, (4) habitual sleep efficiency, (5) sleep disturbances, (6) use of sleep medication, and (7) daytime drowsiness (Multimedia Appendix 6 and 7). The measure has a Cronbach α value of .830 and possesses strong validity [33].

#### Morning and Evening Diary Surveys

We measured self-reported state sleep quality and efficiency with *The Expanded Consensus Sleep Diary for Evening* morning and evening portions, respectively [45] (Multimedia Appendix 3). Research supports the Consensus Sleep Diary as a psychometrically sound daily assessment tool [64].

#### Ambulatory Assessment

We measured physiological trait and state sleep efficiency by recording activity, measured as movement, using the Actiwatch Spectrum Plus (Multimedia Appendix 4). Actigraphy has been found to be a reliable and valid indicator of sleep behavior compared with polysomnography in healthy adults [65].

### Data Reduction and Preprocessing

For all data types, data will be cleaned by examining and applying necessary corrections for outliers, variable normality, and missing data in accordance with standard recommendations [66]. Self-report data will be scored, physiology data will be processed by response channel, and data aggregates will be calculated.

### Data Analysis

Statistical analyses will be performed using R software (R Foundation for Statistical Computing) [67].

#### Preliminary Analyses

The incidence of missing data and technical or compliance deviations will be reported. Descriptive statistics will be presented using frequency tables for categorical variables and means and SDs or SEs of the mean for continuous variables. Pertinent group differences will be tested with chi-square tests, Mann-Whitney *U* tests, 2-tailed *t* tests, or analyses of variance.

Preliminary analyses will address the (1) evaluation of reliability and validity of core outcome measures; (2) evaluation of temporal convergence of core outcome measures based on T1 and T2 individual-difference assessments; (3) evaluation of construct convergence of core outcome measures based on self-reported and physiological state and trait measures; and (4) characterization of the sample regarding demographics and core outcome measures based on self-reported and physiological trait and state measures, including means, subgroups, internal reliability, and temporal variability.

#### Analyses for Testing Core Hypotheses

Preregistered analyses will address the primary objective of testing for ER impairment in those with SB and the secondary objective of testing for mediation pathways in the bidirectional relationships among SB, ER, affect, and sleep.

To address our primary objective, we will test our hypotheses by using SB measures such as self-reported and physiological trait and state SB (Table 1) as predictors and ER measures such as self-reported and physiological trait and state ER (Table 2) as outcomes in targeted contrasts based on the general linear model.

Control analyses will test whether the relationship between SB and ER remains when adjusting for the influences of affective and sleep measures, that is, self-reported and physiological trait and state affect (Table 2) and self-reported trait and state sleep (Table 1). To identify potential control variables, we will test for group differences and associations of SB and ER each with affective and sleep measures. Using the responses from web-based screening surveys, we will additionally test for associations with demographic variables, including sex, age, ethnicity, race, and marital and socioeconomic status. We will include in the primary-objective analyses those variables for which group differences or associations were identified using appropriate statistical methods [68].

To address our secondary objective, we will use cross-sectional and longitudinal mediation analyses on self-reported and physiological trait and state measures of SB, ER, affect, and sleep for contrasting various competing models of directional effects.

## Results

This research received funding in April 2017. Data collection took place from August 2020 to March 2022. A total of 9378 participants were recruited, of whom 237 (2.53%) were eligible and completed the study. Data analysis has not yet started. We expect the first results to be published by the end of 2023.

## Discussion

### Expected Findings

This study seeks to examine the roles of ER, affect, and sleep in individuals with SB and those without through a remote longitudinal observational study. Although existing data on the association between SB and ER are conflicting and limited [14], our aim is to better phenotype ER in SB by (1) testing whether ER is impaired in SB while adjusting for the potential control variables of affect and sleep and (2) testing mediation pathways in the bidirectional relationships among SB, ER, affect, and sleep. We anticipate that the findings from this study will provide insights into the intricate interplay among these factors and their potential implications for understanding and managing SB.

### Strengths and Limitations

This study has several strengths: the longitudinal at-home design enables more representative data collection and ecological validity of the night-to-night variable expression of SB than 1 or 2 nights in a sleep laboratory. This study also involves a large sample size of >200 participants, EMA that eliminates memory bias, and ambulatory assessment of physiological data as objective indicators of SB, ER, and sleep.

This study also has limitations: first, it conducted data collection during the COVID-19 pandemic. The experience of the pandemic likely affected participants’ SB [69], emotion generation and regulation [70], HRV [71], and sleep [72]. Second, this study used a descriptive observational protocol and no experimental manipulation, precluding causal inference. Third, although the remote nature of our study is novel, it presents unique challenges, increasing the risk of noncompliance and technical difficulties. Fourth and last, the EMA surveys may have caused participants to consider and process their emotions more frequently or in different ways than usual, potentially altering their typical SB, ER, affect, or sleep.

### Conclusions

SB is a prevalent oral behavior that, if overexpressed, can be a disorder in susceptible individuals, with a significant negative impact on well-being for those affected [1,6]. The findings of this study may contribute to a better understanding of the relationship among SB, ER, affect, and sleep disturbances. If, as hypothesized, we find maladaptive ER in participants with SB, then this study will represent a meaningful step toward developing treatments for SB through ER intervention. Future studies will need to demonstrate a causal role of impaired ER in SB through extended longitudinal and experimental laboratory studies. We hope that the effort to thoroughly measure SB and ER using gold standard methods and cutting-edge technology will advance the knowledge of SB as well as initiate more effective interventions through ER to alleviate the pain and distress of those affected.

## Appendix

Multimedia Appendix 1 Web-based screening survey.

Multimedia Appendix 2 Ecological momentary assessment software screenshots.

Multimedia Appendix 3 Ecological momentary assessment.

Multimedia Appendix 4 Physiological assessment devices.

Multimedia Appendix 5 Study completion.

Multimedia Appendix 6 Baseline (T1) individual-difference assessment.

Multimedia Appendix 7 Poststudy (T2) individual-difference assessment.

Multimedia Appendix 8 National Institutes of Health peer-review report.

## Abbreviations

ECG: electrocardiography
EMA: ecological momentary assessment
EMG: electromyography
ER: emotion regulation
HRV: heart rate variability
NIH: National Institutes of Health
PROMIS: Patient-Reported Outcomes Measurement Information System
REDCap: Research Electronic Data Capture
RMMA: rhythmic masticatory muscle activity
SB: sleep bruxism

## Data Availability

Study materials, data, and analysis code will be available through an Open Science Framework repository [73].
