# Supplementary material for: The Role of Emotion Regulation, Affect, and Sleep in Individuals With Sleep Bruxism and Those Without: Protocol for a Remote Longitudinal Observational Study
Source: JMIR Res Protoc. 2023 Aug 24;12:e41719. doi: 10.2196/41719 (PMC10485716; doi:10.2196/41719)
Supplement: Multimedia Appendix 1 [file resprot_v12i1e41719_app1.pdf]

Multimedia Appendix 1. Web-Based Screening Survey

1. Web-Based Screening Survey Questions 2

1.1 Initial questions 2

1.2 Study details [after reading all steps involved in study participation] 2

1.3 Participant Information 2

1.4 General Health and Medical History 3

1.5 Questions about behaviors and symptoms pertaining to the mouth and face during sleep .. 4

1.6 Questions about behaviors and symptoms pertaining to the mouth and face during wakefulness 5

1.7 Questions about vision/hearing 7

1.8 Travel plans 7

1.9 Use of tobacco/nicotine products 7

1.10 Use of caffeinated beverages 7

1.11 Psychiatric Condition 8

1.12 Reproductive Health 11

1.13 Sleep 11

1.14 Teeth 13

1.15 Questions pertaining to potential future studies 13

1.16 Completed web-based screening information 14

1. Web-Based Screening Survey Questions

1.1 Initial questions

1. First provide ZIP code.
2. Where did you hear about us?

1.2 Study details [after reading all steps involved in study participation]

1. After reading study steps, do you wish to continue?
2. If eligible for the study, would you be willing to do the following:
    a. Maintain reliable access to the internet?
    b. Have access to a computer with audio and visual capabilities for a web-based study session?
    c. Maintain reliable access to a smartphone?
    d. Be willing and able to fill out a twice-daily paper assessment, if you ever find yourself without access to a smartphone?
    e. Maintain a relatively stable bedtime routine?
    f. Return devices at the end of the study by mail or if the study is terminated?
    g. Refrain from caffeine intake after 12 pm?
    h. Refrain from ANY alcohol intake during your participation in the study?
    i. Refrain from ANY cannabis intake during your participation in the study?

1.3 Participant Information

1. What is your age?
2. What is your sex?
3. What is your weight in pounds?
4. What is your height in feet and inches?
5. Do you have chest hair?
6. What is your marital status?
7. What is your living arrangement?
8. If you have children, how old is the youngest?
9. What is your ethnicity?
10. What is your race [select all that apply]?
11. What is your native language?
12. How do you rate your English proficiency?
13. What is the highest degree you earned?
14. Which of the following best describes your current employment status?
15. Currently, what is your main occupation or job? [Be specific]

16. What is your typical work schedule?
17. Does your work require you to be on call at night?
18. Please choose the option that tells us your approximate total family income per YEAR
19. Are you a U.S. citizen?

1.4 General Health and Medical History

1. Do the following descriptions apply to you?
   a. I don't have any current illnesses or chronic health problems
   b. I have never had any heart problems or cardiovascular conditions, such as heart attack, blood clots, irregular heartbeat, heart failure, congenital heart disease, or any other heart problems
   c. I have never had any lung disease or breathing problems, such as chronic cough, asthma, pneumonia, or any other lung problem
   d. I have never had kidney disease (e.g. kidney failure)
   e. I have never had any joint disease (e.g., lupus, rheumatoid arthritis)
   f. I have never had thyroid disorder
   g. I have never had Chronic Fatigue Syndrome
   h. I have never had any type of cancer
   i. I have never had any movement disorder such as Parkinson's disease, simple or complex tics, Huntington's disease, oromandibular dystonia, or hemifacial spasm
   j. I have never been diagnosed with any major psychological conditions, such as depression, anxiety, or any other mental health issue that impacts my quality of life
   k. I have never had any brain or neurological disorder, such as stroke, cerebral hemorrhage, cerebral atrophy, coma, or other neurological-related conditions
   l. I have never had head trauma, concussion, or brain injury
   m. I have never had seizures (even in the distant past)
   n. I have never had a bowel disorder (e.g., IBS, Crohn's disease)
2. Do you have frequent or intense headaches?
3. Do your headaches usually last more than 4 hours?
4. Do you usually suffer from nausea when you have a headache?
5. Does light or noise bother you when you have a headache?
6. Does your headache limit any of your physical or intellectual activities?
7. How severe was your head trauma, concussion, or brain injury?
8. How many head traumas, concussions, or brain injuries have you had?
9. When did you last suffer a head trauma, concussion, or brain injury? If you are unsure, please enter an approximate date

10. In the past month, did you take any medications, including any over-the-counter medications?

11. Do the medications belong to any of the following categories?

    a. No
    b. Antidepressants, such as Elavil, Trazodone, Prozac, or Zoloft
    c. Anti-anxiety drugs, such as Valium, Xanax, or Buspar
    d. Anti-psychotics, such as Thorazine, Haldol, or Risperidone
    e. Antihypertensives, such as beta blockers or Clonidine
    f. Thyroid medication, such as Thyroxin
    g. Anti-Asthmatics: such as Theophyllin or Clebuterol
    h. Anti-Parkinson drugs, such as L-Dopa, Sinemet, or Requip
    i. Anticonvulsants, such as Dilantin, Tegretol, or Phenobarbital
    j. Headache medicines, such as Cafergot or Imitrex
    k. Stimulants, such as Ritalin or Cylert
    l. Sleep medicines, such as Ambien, Trazodone, Amitriptyline, or Benadryl
    m. Over-the-counter pain relievers, such as Acetaminophen, or Ibuprofen
    n. Oral contraceptives or hormonal treatments
    o. Please list the name, dose, frequency, and approximately how long you have been taking each medication

12. Do you have any chronic pain?

    a. If yes: please list the location(s) of your chronic pain
        i. How would you rate your pain (from no pain to worst pain imaginable)

13. Have you ever been diagnosed with ADHD (Attention Deficit Hyperactivity Disorder)?

14. Do you have any skin allergy, cosmetic allergy, respiratory allergy, or food allergy?

    a. If yes: do you have any food allergies, such as peanut, nut, flour, fish, shellfish allergy?
    b. Do you have any respiratory allergies, such as dust mites, pets, pollen, mold, or particulates?
    c. Do you have any skin allergies, such as contact dermatitis (e.g., nickel, fragrances, preservatives), urticaria (hives), or photo allergy?
    d. Do you have any cosmetic allergies, such as in response to soap, shampoo, moisturizers, deodorants, shaving products, or make-up?

1.5 Questions about behaviors and symptoms pertaining to the mouth and face during sleep

1. During sleep, how frequently do you clench your teeth?
2. During sleep, how frequently do you grind your teeth?
3. During sleep, for how long have you clenched or ground your teeth?

4. During the past 6 months, how frequently have you experienced any of the following symptoms upon awakening?
    a. Sensitive teeth to cold/hot liquid or air
    b. Tension in your teeth
    c. Pain in the jaw joints
    d. Stiffness in your jaw
    e. Sensation of fatigue in your jaw
    f. Hearing or feeling a “click” in your jaw joint that disappears afterwards
    g. Earache
    h. Aching of your temples/headache
    i. Pain in the back of your neck
    j. Back pain

1.6 Questions about behaviors and symptoms pertaining to the mouth and face during wakefulness

1. During waking hours, how frequently do you clench your teeth?
2. During waking hours, how frequently do you grind your teeth?
3. During waking hours, for how long have you clenched or ground your teeth?
4. During the past 6 months, how frequently have you experienced any of the following symptoms towards the end of the day?
    a. Sensitive teeth to cold/hot liquid or air
    b. Tension in your teeth
    c. Pain in the jaw joints
    d. Stiffness in your jaw
    e. Sensation of fatigue in your jaw
    f. Sensation of tightness of your jaw
    g. Sensation of soreness of your jaw
    h. Feeling that your jaw is clenched
    i. Jaw lock or difficulty opening your mouth wide
    j. Feeling as if you had to move your lower jaw to release it
    k. Hearing or feeling a “click” in your jaw joint that disappears afterwards
    l. Earache
    m. Aching of your temples/headache
    n. Pain in the back of your neck
    o. Back pain
5. In the past two weeks (including last night), have you used any kind of treatment to improve your teeth grinding?
    a. Mouth guard/oral splint
    b. Behavioral therapy (e.g., changes to your daily behavior)
    c. Biofeedback/meditation/yoga

d. Medications

e. Other

6. How often do you do each of the following activities, based on the last month? If the frequency of the activity varies, choose the higher option

a. Activities during sleep

i. Clench or grind teeth when asleep, based on any information you may have?

ii. Sleep in a position that put pressure on the jaw (for example, on stomach, on the side?

7. How often do you do each of the following activities, based on the last month? If the frequency of the activity varies, choose the higher option.

a. Activities during waking hours

i. Grind teeth together during waking hours

ii. Clench teeth together during waking hours

iii. Press, touch, or hold teeth together other than while eating (that is, contact between upper and lower teeth)

iv. Hold, tighten, or tense muscles without clenching or bringing teeth together

v. Hold or jut jaw forward or to the side

vi. Press tongue forcibly against teeth

vii. Place tongue between teeth

viii. Bite, chew, or play with your tongue, cheeks, or lips

ix. Hold jaw in rigid or tense position, such as to brace or protect the jaw

x. Hold between the teeth or bite objects such as hair, pipe, pencil, pens, fingers, fingernails, etc.

xi. Use chewing gum

xii. Play musical instrument that involves use of mouth or jaw (for example, woodwind, brass, string instruments)

xiii. Leans with your hand on the jaw, such as cupping or resting the chin in the hand

xiv. Chew food on one side only

xv. Eating between meals (that is, food that requires chewing)

xvi. Sustained talking (for example, teaching, sales, customer service)

xvii. Singing

xviii. Yawning

xix. Hold telephone between your head and shoulders

1.7 Questions about vision/hearing

1. Do you have any uncorrectable visual or auditory impairments?
   a. If yes, please describe
2. Have you ever been diagnosed with astigmatism?
3. Have you been diagnosed with nystagmus?
4. Have you been diagnosed with amblyopia?
5. Have you ever had laser eye surgery?
   a. If yes: when did you have laser eye surgery?
   b. Why did you have laser eye surgery?
6. Do you wear colored contacts or costume contacts?
7. Do you have normal vision without glasses?

1.8 Travel plans

1. Do you have scheduled travel in the next 3 months?
   a. If yes: Do you have scheduled travel in the next 2 months?
      i. If yes: Do you have scheduled travel in the next month?
   b. What is your (soonest) planned date of travel? If you are unsure, please enter an approximate date
2. Did you travel to a different time zone in the past 3 months?
   a. If yes: when was the last day of your trip? If you are unsure, please enter an approximate date

1.9 Use of tobacco/nicotine products

1. Do you smoke or use any tobacco/nicotine products?
   a. If yes: What tobacco/nicotine products have you used?
   b. Currently, how often do you smoke or use tobacco/nicotine products?
2. How often do people smoke or use tobacco/nicotine products inside your home?
3. Outside of your home, are there situations where you are exposed to secondhand smoke (work, in your neighborhood, etc.)?
4. Overall, how often are you exposed to secondhand smoke?

1.10 Use of caffeinated beverages

1. How many cups of caffeinated beverages, such as coffee, soda, tea, or iced tea, do you typically consume in an average day?
2. In the last year, did you drink a lot more caffeine than you used to in order to get the same feeling?
3. In the past year, have you noticed that when you drank the same amount of

caffeine it had less of an effect on you?

4. When you reduced or stopped using caffeine, did you have any of the following (withdrawal symptoms)? If you did not experience any symptoms, please choose “None”
   a. N/A : I do not regularly use caffeine
   b. N/A : I have not reduced or stopped my caffeine usage
   c. Nervousness or anxiety
   d. Restlessness
   e. Stomachaches, nausea, or vomiting
   f. Headaches
   g. Drowsiness
   h. Sadness/depression
   i. Fatigue
   j. Sluggishness or feeling slowed down
   k. None: I experienced no symptoms

1.11 Psychiatric Condition

1. Do you currently have or have you ever been diagnosed with any psychiatric condition
   a. Please describe
2. Were you ever:
   a. Depressed or down, or felt sad, empty or hopeless most of the day, nearly every day?
   b. Much less interested in most things or much less able to enjoy the things you used to enjoy most of the time?
3. Have you ever had a period of time when you were feeling ‘up’, ‘high’, ‘hyper’, or so full of energy or full of yourself that you got into trouble, or that other people thought you were not your usual self? (Do not consider times when you were intoxicated on drugs or alcohol)
4. Have you ever been persistently irritable, for several days, so that you had arguments or verbal or physical fights, or shouted at people outside your family?
5. Have you or others noticed that you have been more irritable or over reacted, compared to other people, even in situations that you felt were justified?
6. Have you, on more than one occasion, had spells or attacks when you suddenly felt anxious, very frightened, uncomfortable or uneasy, even in situations where most people would not feel that way?
7. Did the spells surge to a peak within 10 minutes of starting?
8. Do you feel anxious or uneasy in places or situations where help might not be available or escape might be difficult if you had a panic attack or panic-like or

embarrassing symptoms?

a. Being in a crowd
b. Standing in line
c. Being in an open space
d. Crossing a bridge
e. Being in an enclosed space away from home
f. Using public transportation
g. N/A

9. In the past month, did you have persistent fear and significant anxiety at being watched, being the focus of attention, or of being humiliated or embarrassed or rejected? (this includes things like speaking in public, eating in public or with others, writing while someone watches, performing in front of others or being in social situations)

10. In the past month, have you been bothered by recurrent thoughts, impulses, or images that were unwanted, distasteful, inappropriate, intrusive, or distressing? (For example, the idea that you were dirty, contaminated or had germs or fear of contaminating others, or fear of harming someone even though it disturbs or distresses you, or fear you would act on some impulse, or fear or superstitions that you would be responsible for things going wrong, or obsessions with sexual thoughts, images or impulses, or religious obsessions.)

a. If yes: in the past month, did you try to suppress these thoughts, impulses, or images or to neutralize or to reduce them with some other thought or action?

11. In the past month, did you feel driven to do something repeatedly in response to an obsession or in response to a rigid rule, like washing or cleaning excessively, counting or checking things over and over, or repeating or arranging things, or other superstitious rituals?

a. If yes: are these rituals done to prevent or reduce anxiety or distress or to prevent something bad from happening and are they excessive or unreasonable?

12. Have you ever experienced or witnessed or had to deal with an extremely traumatic event that included actual or threatened death or serious injury or sexual violence to you or someone else?

Examples of traumatic events include: serious accidents, sexual or physical assault, a terrorist attack, being held hostage, kidnapping, fire, discovering a body, war, or natural disaster, witnessing the violent or sudden death of someone close to you, or a life threatening illness.

a. If yes: Starting from the traumatic event, did you repeatedly re-experience the event in an unwanted mentally distressing way (such as recurrent dreams related to the event, intense recollections or memories, or flashbacks or as if the event was recurring) or did you have intense physical or psychological reactions when you were reminded of the event or exposed to a similar event?

13. In the past 12 months have you had 3 or more alcoholic drinks within a 3-hour period on 3 or more occasions?

14. In the past 12 months, did you take any of the drugs listed below more than once, to get high, to feel elated, to get a buzz, or to change your mood?

   a. Amphetamines
   b. Speed, crystal meth
   c. Dexedrine, Ritalin
   d. Cocaine, crack
   e. LSD, mescaline
   f. Steroids, GHB
   g. Morphine, methadone
   h. PCP, angel dust, ecstasy
   i. Valium, Xanax
   j. Opium, Demerol
   k. MDA, MDMA
   l. Diet pills, Rush
   m. Ativan
   n. Codeine
   o. Ketamine
   p. THC, marijuana, cannabis, hashish
   q. Barbiturates
   r. Percodan, OxyContin, Vicodin
   s. Inhalents glue, ether
   t. Heroin
   u. Other
   v. None

15. What was your lowest weight in the past 3 months?
16. In the past 3 months, did you have eating binges or times when you ate a very large amount of food within a 2-hour period?
   a. If yes: during these binges did you feel that your eating was out of control?
17. Were you excessively worried about routine things in the past 6 months?
   a. If yes: are these anxieties or worries present most days?
18. Have you received psychotherapy in the past year?
   a. If yes: in treatment or therapy, what did you do? What do you remember from it?
19. Have you ever participated in cognitive behavioral therapy before?
20. In treatment or therapy, did you ever learn skills or implement skills at home?
21. In treatment or therapy, did you have homework?
22. In treatment or therapy, how often did you try to implement these skills?

23. In treatment or therapy, did you do thought logs?

1.12 Reproductive Health

1. If applicable, do you have a regular menstrual cycle?
   a. N/A (I'm a male who does not menstruate)
   b. Yes (I have regular periods)
   c. No (I am pregnant, my last pregnancy ended within the past 2 months, or I'm breastfeeding)
   d. No (I am going through menopause)
   e. No (my periods are irregular)
   f. N/A (I do not have periods)
2. Menstrual Health:
   a. Typically, how long does your period last?
   b. Typically, how often do you have your period?
   c. When was the first day of your last menstrual period?
   d. Do you experience any of the following around your periods? (mood swings, fatigue, concentration difficulty, depression, nervousness, irritability, nausea, swelling of ankles, feet, or hands, weight gain, No)
3. Do you currently use any hormonal contraception or hormone-replacement therapy?
   a. If yes: which hormonal contraception or hormone-replacement therapy do you currently use (type and name)?
4. Do you plan to become pregnant within the next 3 months?

1.13 Sleep

1. What time do you usually wake up in the morning on a work day?
2. What time do you usually go to bed at night on a work day?
3. What time do you usually wake up in the morning on a weekend?
4. What time do you usually go to bed at night on a weekend?
5. Is your sleep regularly disrupted by any of the following? (your bed partner, light, the need to care for another [i.e. children, elderly parent, etc.], worries, pet(s), temperature, pain/discomfort, noise, the need to urinate, bed/mattress, other, N/A)
6. What position do you sleep in? Please check all that apply (back, left side, right side, stomach)
7. Please describe any sleeping arrangement or special environment you may have in or near your bed, for example, using pillows, towels, heat pads, temperature control, or other props
8. Last night

a. How long did you sleep? If unsure, please approximate
b. When did you go to bed? If unsure, please approximate
c. When did you wake up? If unsure, please approximate

9. 2 nights ago
   a. How long did you sleep? If unsure, please approximate
   b. When did you go to bed? If unsure, please approximate
   c. When did you wake up? If unsure, please approximate
10. 3 nights ago
    a. How long did you sleep? If unsure, please approximate
    b. When did you go to bed? If unsure, please approximate
    c. When did you wake up? If unsure, please approximate
11. 4 nights ago
    a. How long did you sleep? If unsure, please approximate
    b. When did you go to bed? If unsure, please approximate
    c. When did you wake up? If unsure, please approximate
12. Do you currently use any sleep tracking devices or apps?
13. One hears about "morning" and "evening" types of people. Which one of these types do you consider yourself to be? (definitely more of a morning type, rather more of a morning type, rather more of an evening type, definitely more of an evening type)
14. Do you snore loudly (louder than talking or loud enough to be heard through closed doors)?
15. Do you often feel tired, fatigued, or sleepy during daytime?
16. Has anyone observed you stop breathing during your sleep?
17. Do you have or are you being treated for high blood pressure?
18. Is your neck circumference larger than 16 inches (41 cm)?
19. Do you use a sleep apnea treatment device when you sleep? (includes CPAP machines)
20. Do you have any problems sleeping?
21. Do you usually sleep more than 9 hours but feel unrefreshed when you get up in the morning?
22. Do you often take long naps during the day?
23. Do you find yourself so sleepy during the day that you have to struggle to stay awake or doze off unintentionally?
24. Do you have irresistible attacks of sleep?
25. Do you snore or wake up gasping for air?
26. Do you have a sleep/wake schedule that is unusual to you or different from the sleep/wake pattern of other people?
27. Do you ever get an unpleasant feeling in your legs or have an urge to move your legs when you are resting?
28. Do you ever awaken from nighttime sleep with terribly frightening dreams?

29. Have you ever had (or has anyone ever told you about) abrupt awakenings from sleep beginning with a loud scream?
30. Do you have episodes of arising from bed during sleep and walking about?
31. Have you ever "acted out" your dreams while asleep?
32. Have you ever injured yourself or a bed partner during sleep?

1.14 Teeth

1. How many natural teeth do you have per quadrant?
2. Do you have gum recession?
3. Do you have any cracked or worn down teeth?
4. Has your dentist ever told you that you might be grinding your teeth?
5. Have you ever been diagnosed with any oral disease (e.g., aphthous ulcer, pockets, inflammation, cysts)?
6. Are you currently diagnosed and undergoing any treatment for periodontal disease (e.g. symptoms include swollen, red, and tender gums)?
7. Are you currently under active orthodontic braces/headgear treatment or tooth repair?
8. Do you wear a retainer, a mouthguard, Invisalign, or an oral splint?
9. Do you have any removable partial dentures?
10. Would you be willing and able to refrain from wearing your retainer, mouthguard, Invisalign, or oral splint for the duration of the study?
11. In the last 30 days, did your jaw lock or catch, even for a moment, so that it would not open all the way?
12. In the last 30 days, when you opened your mouth wide, did your jaw lock or catch, even for a moment, so that you could not close it from this wide open position?
13. Are you able to open up your mouth wide more than 3 cm?
14. Have you ever had any history of trauma to your jaw?
15. In the last 30 days have you had any clicking sounds when you moved or used your jaw?
16. In the last 30 days have you had any grating sound when you moved or used your jaw?
17. In the last 30 days, have you had any difficulty opening your mouth?
18. In the last 30 days, have you had pain or stiffness in your jaw?

1.15 Questions pertaining to potential future studies

1. Do you have locs, braids (that you are not willing to unbraid) , hair extensions (that you are not willing to remove), or a sensitive scalp that would prevent us from putting sensors on your head?
2. What is your dominant hand (right hand, left hand, ambidextrous - both hands or

no preference)?

3. Please indicate which hand you predominantly use for each of the following activities:
    a. Writing, throwing, toothbrushing, using a spoon
4. Have you been diagnosed with color blindness?

1.16 Completed web-based screening information

1. Please provide your email
2. May we contact you by phone?
3. What is the next phone number with which to reach you?
4. Do you use an Android or an iPhone?
5. Do you have one or more alternate phone numbers?
6. What would be the best time to reach you during the week?
