# Supplementary material for: The Role of Emotion Regulation, Affect, and Sleep in Individuals With Sleep Bruxism and Those Without: Protocol for a Remote Longitudinal Observational Study
Source: JMIR Res Protoc. 2023 Aug 24;12:e41719. doi: 10.2196/41719 (PMC10485716; doi:10.2196/41719)
Supplement: Multimedia Appendix 2 [file resprot_v12i1e41719_app2.pdf]

Multimedia Appendix 2: Ecological Momentary Assessment Software Screenshots

1. Sample question on negative affect as viewed on a smartphone 2

2. Sample question on the “distract” emotion regulation strategy as viewed by a desktop............ 3

3. Sample question and response from Morning Diary as viewed by a smartphone 4

4. Sample question and response from Morning Diary as viewed by a desktop 5

5. Sample follow-up questions on emotion regulation behaviors as viewed by a desktop............. 6

1. Sample question on negative affect as viewed on a smartphone

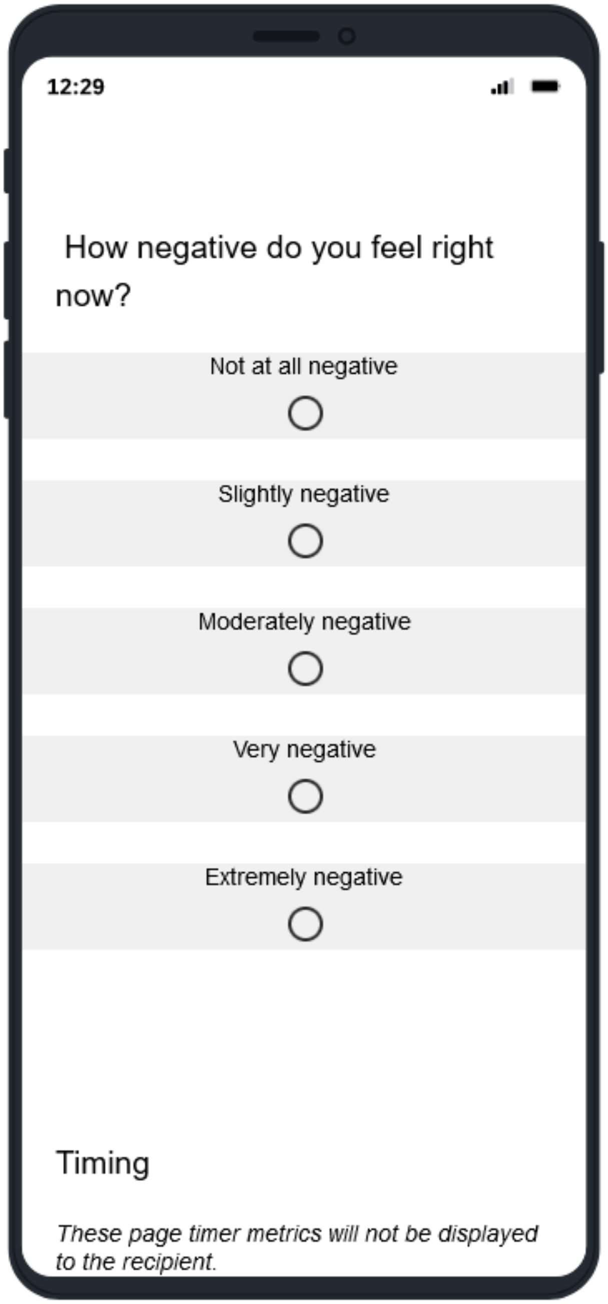

2. Sample question on the “distract” emotion regulation strategy as viewed by a desktop

Since the situation happened, I tried **focusing on something unrelated to the situation** with the goal of changing my negative emotion.

Not at all	A little	A moderate amount	A lot	A great deal
○	○	○	○	○

3. Sample question and response from Morning Diary as viewed by a smartphone

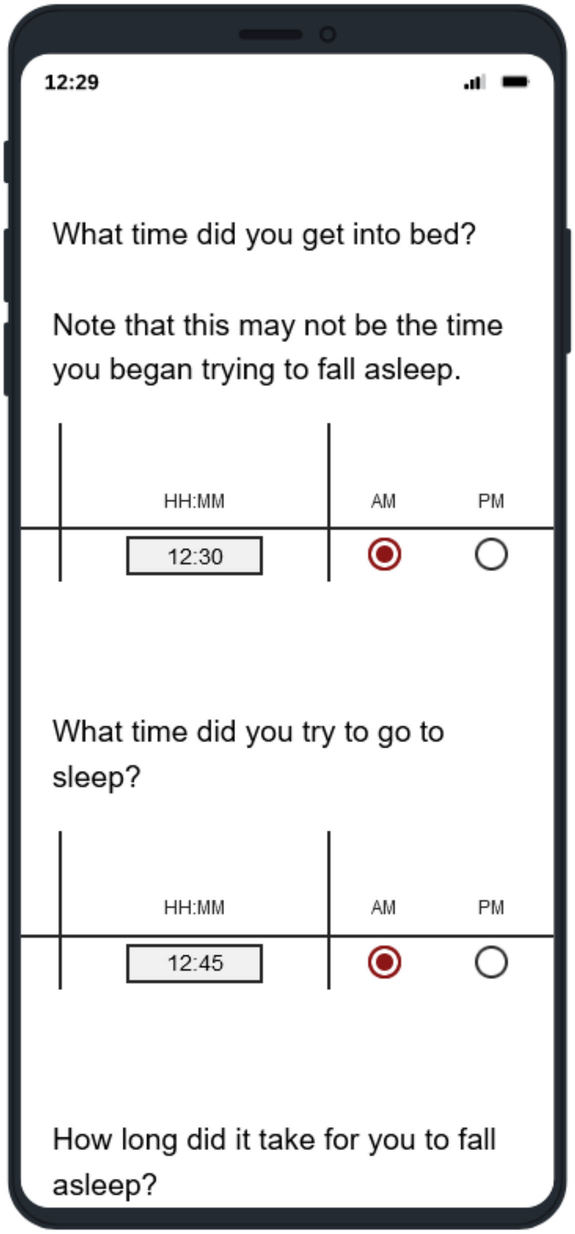

4. Sample question and response from Morning Diary as viewed by a desktop

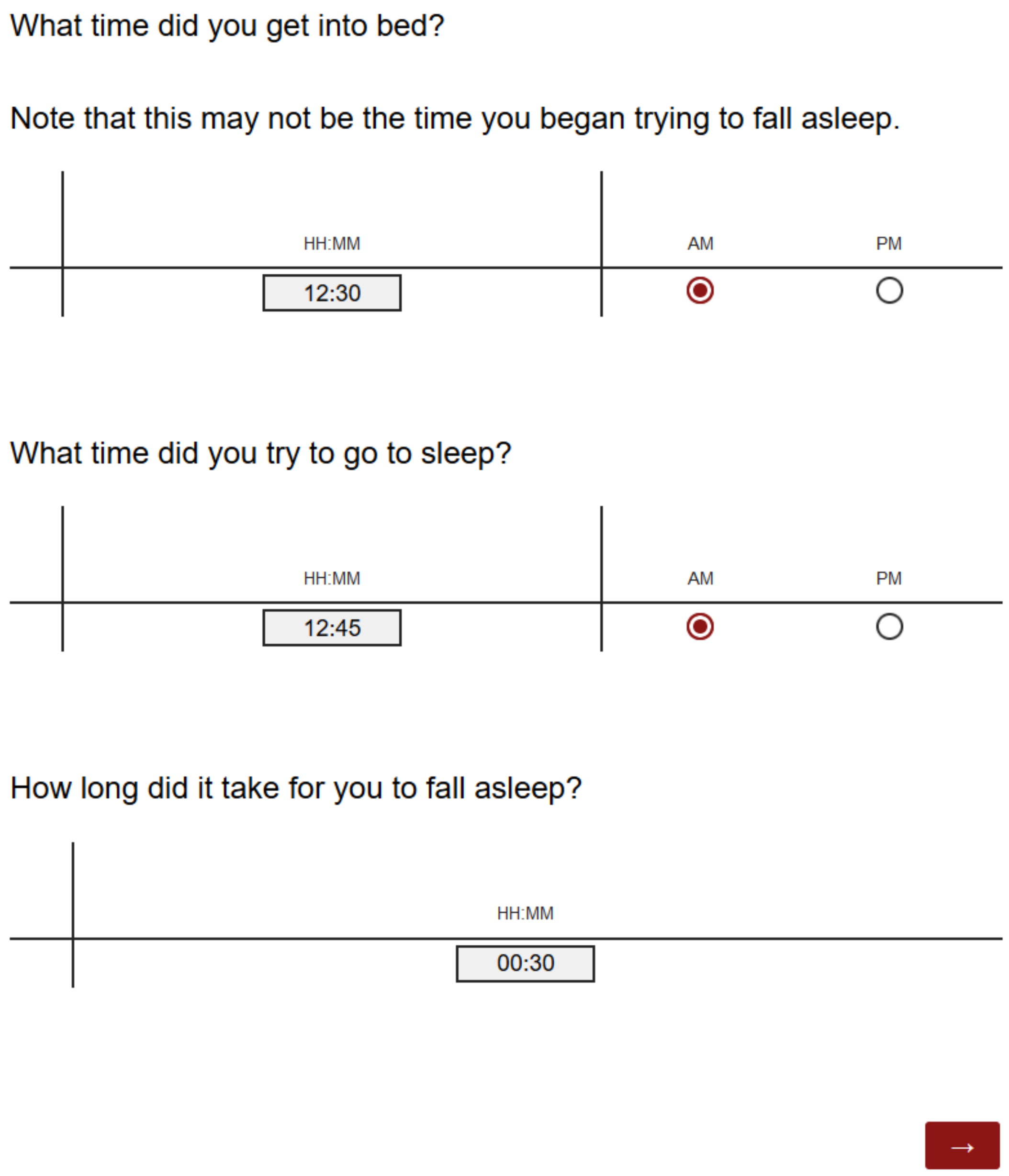

5. Sample follow-up questions on emotion regulation behaviors as viewed by a desktop

Please answer the following questions.

Since after you originally reported the event 24 hours ago, did you or did you not consume alcohol, tobacco, marijuana, or other substances (excluding caffeine)?

- (●) Yes, I did
- ( ) No, I did not

Since after you originally reported the event 24 hours ago, did you or did you not eat food?

- ( ) Yes, I did
- (●) No, I did not

Since after you originally reported the event 24 hours ago, did you or did you not spend time with others?

- (●) Yes I did
- ( ) No I did not
