# Supplementary material for: The Role of Emotion Regulation, Affect, and Sleep in Individuals With Sleep Bruxism and Those Without: Protocol for a Remote Longitudinal Observational Study
Source: JMIR Res Protoc. 2023 Aug 24;12:e41719. doi: 10.2196/41719 (PMC10485716; doi:10.2196/41719)
Supplement: Multimedia Appendix 3 [file resprot_v12i1e41719_app3.pdf]

Multimedia Appendix 3. Ecological Momentary Assessment

1. EMA training 1

2. Ecological Momentary Assessment 2

2.1. Morning Diary Survey 3

2.2. Daytime Survey 4

2.3. Evening Diary Survey 5

References 7

1. EMA training

The EMA training serves as a self-paced training for the participant to learn how to complete the EMA surveys during the 14-day ambulatory assessment on their phones. The EMA training, created by our research team, consists of instructional texts, video clips, as well as multiple choice and open-ended comprehension questions. Participants will complete the training in a self-paced manner, taking approximately 30 minutes. The training consists of 49 pages, 32 brief videos, and 123 questions. Through a series of brief videos, research assistants taught participants how to answer the questions asked in the morning diary, evening diary, and daytime surveys.

First, the videos introduce the EMA training and surveys, describing how and when to complete them as well as their importance to the study. Second, they discuss how to answer

questions found in the EMA surveys. Third, they demonstrate how to answer the optional open-ended question, found in both the morning and evening diaries, that asks whether they have any concerns or want to elaborate on previous answers. Throughout the training, participants will answer 2 types of questions: questions for practice purposes (these will be the same items found in the morning diary, evening diary, and daytime survey) and questions for comprehension assessment (these will be items regarding how and when to answer the surveys).

2. Ecological Momentary Assessment

EMA surveys were delivered using a web application built by our research team. The web application allows research assistants to enroll new participants and create a schedule for when EMA text message reminders with the survey link are delivered to them. This system was hosted on a server (Amazon Web Services, Inc., Seattle, WA) that provides an on-demand cloud computing platform and APIs delivered through an automated text messaging system (Twilio, San Francisco, CA). The web application sent API request to Twilio at the scheduled times, and Twilio sent pre-defined EMA text messages to participants.

Participants were instructed to complete the morning survey 15 minutes after getting out of bed, the evening survey 30–40 minutes before going to bed, and the 3 daytime surveys as soon as possible after they receive the text messages. The 3 daytime surveys were spaced at least 3 hours apart. All 5 surveys will be set to arrive within a 30-minute window (jittered +/-15 minutes from the scheduled time) to prevent the timing of the assessment from being a confounding factor. Participants were given a maximum of 3 hours to complete the surveys and received a follow-up text after ~30 minutes if no response was received. If the research team didn't receive responses to the EMA surveys for more than 24 hours, the research team reached out to the

participant to ensure that there are not any technical issues keeping them from completing the EMAs.

2.1. Morning Diary Survey

The morning diary survey includes questions pertaining to:

1. SB: teeth grinding and jaw pain
2. ER: a participant's degree of use and success of pre-sleep ER
3. Affect: a participant's degree of current positive and negative affect
4. Sleep: Consensus Sleep Diary – E morning portion; Carney et al., 2012

Morning Diary Survey items:

- To your knowledge, did you or did you not grind or clench your teeth last night?
- How much jaw pain or soreness are you feeling right now?
    - If you are not experiencing jaw pain or soreness, please select "none at all."
- Did you wear any of the following oral appliances last night?
- As you tried to fall asleep last night, how much did you try to change your feelings?
- As you tried to fall asleep last night, how successful were you at changing your feelings?
- Did you experience any issues with the Sleep Buddy last night (ie, it fell off, the blue light didn't flash when you put it in the docking station this morning, etc.)?
    - Please describe the issue that occurred: Comments, anything to note.
- If you had an unusual night or if anything affected your sleep, please describe below.
- Were there any concerns or problems that came up while taking this survey? (For example: misspellings, tapping the wrong button, or typing the wrong time) If so, please explain or make corrections below:

2.2. Daytime Survey

The daytime survey asks participants specifically to think about a negative event that occurred since the last survey (within 3 hours) and includes questions pertaining to:

1. SB: N/A
2. ER: ER strategies they utilized after the event (cognitive reappraisal, attentional distraction, expressive suppression) and their ER success
3. Affect: a participant's degree of current positive and negative affect; and
4. Sleep: N/A.

If participants couldn't think of a recent negative event, they were instructed to complete the survey with respect to a negative event within the past year in order to not differentially affect participants.

Daytime Survey items:

- How positive do you feel right now?
- How negative do you feel right now?
- Since your last survey, please recall and describe a situation when you felt the most negative. It may have been caused by something you thought about or remembered, or a situation that happened.
- Please respond to each statement according to how much you <u>tried</u> to engage in the following behaviors with the goal of changing your negative emotion regarding the situation described below.
- Since the situation happened, I tried changing the way I thought about the situation with the goal of changing my negative emotion.
- Since the situation happened, I tried focusing on something unrelated to the situation with

the goal of changing my negative emotion.

- Since the situation happened, I tried to reduce my outward expression of the emotion.
- Since the situation happened, if you used any of the previously mentioned strategies to reduce your negative emotion, how successful were you at doing so?
- From the past year, please recall and describe a situation when you felt negatively. It may have been caused by something you thought about or remembered, or a situation that happened.
- If you are wearing a CardiacScout heart monitor, please open the app on your phone and reconnect the device if necessary. If you encounter problems connecting, please contact homesleep@stanford.edu.

2.3. Evening Diary Survey

The evening diary survey includes questions pertaining to:

1. SB: teeth behaviors throughout the day adapted from the Oral Behavior Checklist [1]
2. ER: N/A
3. Affect: a participant's degree of current positive and negative affect as well as past-day positive and negative affect and perceived stress
4. Sleep: Consensus Sleep Diary – E, evening portion) [2]

Evening Diary Survey items:

- How positive do you feel right now?
- How negative do you feel right now?
- Overall, how positive did you feel today?

- Overall, how negative did you feel today?
- Overall, how stressed did you feel today?
- Please rate how frequently you engaged in the following behaviors <u>since waking up this morning:</u>
    - Press, touch, or hold teeth together other than while eating (that is, contact between upper and lower teeth)
    - Hold, tighten, or tense muscles without clenching or bringing teeth together
    - Clench teeth together during waking hours
    - Press tongue forcibly against teeth
- Did you or did you not remove the Actiwatch today? If you did, please put it back on.
- Please indicate at what time(s) you removed the ActiWatch today and for how long it was removed each time:
- Did you or did you not remove the CardiacScout today? If so, please put it back on.
- Please indicate at what time(s) you removed the [MultiVitals Monitor] today and for how long it was removed each time:
- Comments, anything to note.
- If you had an unusual day or if anything affected your experience today, please describe below.
- Were there any concerns or problems that came up while taking the surveys today? (For example: misspellings, tapping the wrong button, or typing the time or wrong dose of a medication)

References

1. Ohrbach R, Beneduce C, Markiewicz M, McCall Jr W. Psychometric properties of the oral behaviors checklist: preliminary findings. The International Association for Dental Research. 2004. URL: https://iadr.abstractarchives.com/abstract/ 2004Hawaii-42340/psychometric-properties-of-the-oral-behaviors-checklist-preliminary-findings [accessed 2023-07-17]
2. Carney CE, Buysse DJ, Ancoli-Israel S, Edinger JD, Krystal AD, Lichstein KL, et al. The consensus sleep diary: standardizing prospective sleep self-monitoring. Sleep 2012 Feb 01;35(2):287-302 [FREE Full text] [doi: 10.5665/sleep.1642] [Medline: 22294820]
