# Supplementary material for: The Role of Emotion Regulation, Affect, and Sleep in Individuals With Sleep Bruxism and Those Without: Protocol for a Remote Longitudinal Observational Study
Source: JMIR Res Protoc. 2023 Aug 24;12:e41719. doi: 10.2196/41719 (PMC10485716; doi:10.2196/41719)
Supplement: Multimedia Appendix 4 [file resprot_v12i1e41719_app4.pdf]

Multimedia Appendix 4. Physiological Assessment Devices

1. EMG Butler GrindCare Patch Sensor Device for a Physiological Index of SB 1

2. ECG sensor for a Physiological Index of ER... 2

3. Actiwatch Spectrum Plus for a Physiological Index of Sleep 3

References.. 4

1. EMG Butler GrindCare Patch Sensor Device for a Physiological Index of SB

We obtained an objective, physiological index of SB in terms of RMMA using the portable, single-channel EMG Butler GrindCare patch sensor device (GC401C, Sunstar Suisse SA, Etoy, Switzerland) [1]. The device attaches to a pre-gelled adhesive pad (Sunstar Suisse SA) to either the left or right temple. It records EMG activity over the anterior temporalis muscle by galvanic tripolar electrodes to quantify grinding and clenching activity. Recording from the anterior temporalis muscle has been shown to provide comparable results for RMMA assessment as recording from the masseter muscles [2]. The device runs an on-board signal recognition algorithm, which automatically detects types of RMMA as defined by Lavigne and colleagues [3]. A moving-average algorithm compares the EMG amplitude to the estimated background level and scores events when the EMG signal exceeds the amplitude of the background noise by 3 or more times for a duration of ≥ 0.25 s (cf. RMMA detection rules) [3,4]. Based on these data, the device automatically derives and saves the number of RMMA events per hour of recording

(grinds per hour, GPH), total number of RMMA events per recording period, and sleep duration per recording period. After use, the device is placed into a docking station for charging. The device stores the nightly data on the docking station via Bluetooth. The research team performs data download from the docking station via the GrindCare mobile app (Sunstar Group, Etoy, Switzerland) to a lab computer for storage and future analysis upon the return of the device after the 14-day ambulatory assessment. The device's feature to issue electrical impulses to lower the EMG activity is turned off before the device was mailed to the participants.

2. ECG sensor for a Physiological Index of ER

We obtained continuous ECG using a medical-grade, wearable and reusable sensor (VS-US4 – ECG, VivaLink Inc., Campbell, CA). It utilizes a 3-electrode configuration to collect a single-lead ECG at 128 Hz and a 3-axis accelerometer data at 5 Hz [5]. The system additionally outputs measurements of heart rate, respiratory rate, calorific energy expenditure, activity, and stress. Participants continuously wear the device on the chest above the pectoralis major. It can be worn during exercise or showering given that the device is shock and water resistant. The device must be removed to charge for about 4 hours every 3 days (72 hours). These charging periods, as well as periods in which participants engage in activities that involve being submerged in water for more than 30 minutes, such as swimming, were the only times that participants removed the device. It allows for continuous streaming and 72-hour cache data. Machine-to-Machine API supports the device's automatic Bluetooth connection with the Multi-Vital-Monitor mobile app (v2.0.1, VivaLink, Campbell, CA) to capture data and upload it to the VivaLink Cloud server. The research team performed retrospective data file download from the Cloud (low energy wireless technology; IEEE 802.15.1).

3. Actiwatch Spectrum Plus for a Physiological Index of Sleep

We recorded activity (movement) for estimating nightly sleep efficiency and habitual sleep patterns as well as rest and activity periods during the day using the Actiwatch Spectrum Plus (Philips Respironics Inc., Eindhoven, The Netherlands). This device collects rest–activity data via an accelerometer (piezoelectric accelerometer; sensitivity of 0.025 G-force; 32-Hz sampling rate). Data is collected in 60-second epochs. Participants continuously wear the watch device on their non-dominant wrists while sleeping and awake, as it has sufficient battery life for 14-day monitoring and is shock and water resistant. To allow correct identification of time in bed, the actigraphy device has an "event marker" that participants push to mark time in and out of bed [6]. To define sleep periods, participants were instructed to push an event mark button on the actigraphy device when they intended to sleep or nap and upon waking up. Data download from the actigraphy device to the lab computer was accomplished through Philips Actiware software (Philips, Eindhoven, The Netherlands). Actigraphy has been demonstrated to produce clinically reliable and valid measurements of daytime activity and sleep quality [7-9] using motion, light, and user input. Specifically, the Actiwatch 2 has been validated against the gold-standard of PSG, demonstrating high sensitivity (to detect sleep; 0.97), slightly poorer specificity (to detect wake; 0.77), and overall high accuracy (0.90) [10], and this is consistent with other actigraph models and scoring algorithms used across healthy adults [11,12].

References

1. Thymi, M., Shimada, A., Lobbezoo, F., & Svensson, P. (2019). Clinical jaw-muscle symptoms in a group of probable sleep bruxers. *Journal of Dentistry, 85*, 81-87.
2. Koyano, K., Tsukiyama, Y., Ichiki, R., & Kuwata, T. (2008). Assessment of bruxism in the clinic. *Journal of Oral Rehabilitation, 35(7)*, 495-508.
3. Lavigne, G. J., Rompre, P. H., & Montplaisir, J. Y. (1996). Sleep bruxism: validity of clinical research diagnostic criteria in a controlled polysomnographic study. *Journal of Dental Research, 75(1)*, 546-552.
4. Dreyer, P., Yachida, W., Huynh, N., Lavigne, G. J., Haugland, M., Svensson, P., & Castrillon, E. E. (2015). How close can single-channel EMG data come to PSG scoring of rhythmic masticatory muscle activity. *Journal of Dental Sleep Medicine*, *2(04)*, 147-156.
5. Li, H., & Boulanger, P. (2021). An Automatic Method to Reduce Baseline Wander and Motion Artifacts on Ambulatory Electrocardiogram Signals. *Sensors, 21(24)*, 8169.
6. Martin, J. L., & Hakim, A. D. (2011). Wrist actigraphy. Chest, 139(6), 1514-1527.
7. Cheung, J., Leary, E. B., Lu, H., Zeitzer, J. M., & Mignot, E. (2020). PSG Validation of minute-to-minute scoring for sleep and wake periods in a consumer wearable device. *PLoS One, 15(9)*, e0238464.
8. Joyce, D. S., Zele, A. J., Feigl, B., & Adhikari, P. (2020). The accuracy of artificial and natural light measurements by actigraphs. *Journal of Sleep Research, 29(5)*, e12963.
9. Vitale, J. A., Banfi, G., Tivolesi, V., Pelosi, C., Borghi, S., & Negrini, F. (2021). Rest-activity daily rhythm and physical activity levels after hip and knee joint replacement: The role of actigraphy in orthopedic clinical practice. *Chronobiology International, 38(12)*, 1692-1701.

10. Meltzer, L. J., Walsh, C. M., Traylor, J., & Westin, A. M. (2012). Direct comparison of two new actigraphs and polysomnography in children and adolescents. *Sleep, 35(1)*, 159-166.

11. Aili, K., Åström-Paulsson, S., Stoetzer, U., Svartengren, M., & Hillert, L. (2017). Reliability of actigraphy and subjective sleep measurements in adults: the design of sleep assessments. *Journal of Clinical Sleep Medicine, 13(1)*, 39-47.

12. Sadeh, A. (2011). The role and validity of actigraphy in sleep medicine: an update. *Sleep Medicine Reviews*, *15(4)*, 259-267.
