# Supplementary material for: The Role of Emotion Regulation, Affect, and Sleep in Individuals With Sleep Bruxism and Those Without: Protocol for a Remote Longitudinal Observational Study
Source: JMIR Res Protoc. 2023 Aug 24;12:e41719. doi: 10.2196/41719 (PMC10485716; doi:10.2196/41719)
Supplement: Multimedia Appendix 5 [file resprot_v12i1e41719_app5.pdf]

Multimedia Appendix 5. Study Completion

1. Return Mailing of Physiology Devices 1

2. Monetary Participant Compensation... 1

3. Educational Participant Compensation 2

1. Return Mailing of Physiology Devices

After the study team received the equipment/devices, a research assistant exported, saved, and checked the data both from the participant's RMMA device using the corresponding mobile app and the participant's actigraphy device, using the corresponding computer software. The research assistant also established the count of missing EMA survey responses for the participant, if any, and double checked that all the participant's ECG data files collected by the ECG device have been downloaded from the ECG data Cloud server. All data is currently stored on a local data server.

2. Monetary Participant Compensation

Participants received a maximum compensation of $80 for each component of the study that they completed:

- $15 for the first individual differences assessment
- $10 for the 2 video call sessions
- $10 for completing 80% of the EMA surveys
- An additional $10 for completing 90% of the EMA surveys

- A $30 bonus for continuously wearing the devices throughout the 14-day ambulatory assessment
- $5 for the second individual differences assessment

3. Educational Participant Compensation

Example Sleep Report:

Stanford Home Sleep Center

Your Sleep Time Activity and Personality Survey Data Results

PID: 0000

Disclaimer* The results in this summary packet are not diagnostic. Each person's sleep patterns vary.

Nightly Activity

This section shows a compilation of your self-recorded 2-week sleep activity data.

- **Night 1**
  - Time you went to bed: 22:00
  - Time you tried to fall asleep: 22:05
  - Amount of time it took you to fall asleep: 5 min
  - # of awakenings at night: 1
  - Duration of awakening: 5 min
  - Final time you woke up: 06:00
  - Time you got up from bed: 06:05

- Duration of total sleep: 7 hours 45 min

- **Night 2**
  - Time you went to bed: 22:05
  - Time you tried to fall asleep: 22:10
  - Amount of time it took you to fall asleep: 5 min
  - # of awakenings at night: 1
  - Duration of awakening: 10 min
  - Final time you woke up: 06:05
  - Time you got up from bed: 06:10
  - Duration of total sleep: 7 hours 40 min
- **Night 3**
  - Time you went to bed: 22:30
  - Time you tried to fall asleep: 22:40
  - Amount of time it took you to fall asleep: 3 min
  - # of awakenings at night: 0
  - Duration of awakening: 0 min
  - Final time you woke up: 06:30
  - Time you got up from bed: 06:40
  - Duration of total sleep: 7 hours 47 min
- **Night 4**
  - Time you went to bed: 22:15
  - Time you tried to fall asleep: 22:20
  - Amount of time it took you to fall asleep: 15 min

- # of awakenings at night: 1
- Duration of awakening: 10 min
- Final time you woke up: 06:45
- Time you got up from bed: 06:45
- Duration of total sleep: 8 hours 0 min

- **Night 5**
  - Time you went to bed: 22:00
  - Time you tried to fall asleep: 22:10
  - Amount of time it took you to fall asleep: 10 min
  - # of awakenings at night: 1
  - Duration of awakening: 15 min
  - Final time you woke up: 06:25
  - Time you got up from bed: 06:30
  - Duration of total sleep: 7 hours 50 min
- **Night 6**
  - Time you went to bed: 23:40
  - Time you tried to fall asleep: 23:50
  - Amount of time it took you to fall asleep: 5 min
  - # of awakenings at night: 0
  - Duration of awakening: 2 min
  - Final time you woke up: 07:00
  - Time you got up from bed: 07:05
  - Duration of total sleep: 7 hours 3 min

- **Night 7**
  - Time you went to bed: 23:30
  - Time you tried to fall asleep: 23:35
  - Amount of time it took you to fall asleep: 0 min
  - # of awakenings at night: 0
  - Duration of awakening: 0 min
  - Final time you woke up: 07:25
  - Time you got up from bed: 07:30
  - Duration of total sleep: 7 hours 50 min
- **Night 8**
  - Time you went to bed: 22:45
  - Time you tried to fall asleep: 22:50
  - Amount of time it took you to fall asleep: 4 min
  - # of awakenings at night: 1
  - Duration of awakening: 7 min
  - Final time you woke up: 06:50
  - Time you got up from bed: 07:00
  - Duration of total sleep: 7 hours 49 min
- **Night 9**
  - Time you went to bed: 21:05
  - Time you tried to fall asleep: 21:10
  - Amount of time it took you to fall asleep: 0 min
  - # of awakenings at night: 1

  - Duration of awakening: 5 min
  - Final time you woke up: 05:00
  - Time you got up from bed: 05:05
  - Duration of total sleep: 7 hours 45 min
- **Night 10**
  - Time you went to bed: 21:30
  - Time you tried to fall asleep: 22:00
  - Amount of time it took you to fall asleep: 30 min
  - # of awakenings at night: 1
  - Duration of awakening: 2 min
  - Final time you woke up: 08:00
  - Time you got up from bed: 08:00
  - Duration of total sleep: 9 hours 28 min
- **Night 11**
  - Time you went to bed: 23:50
  - Time you tried to fall asleep: 23:55
  - Amount of time it took you to fall asleep: 0 min
  - # of awakenings at night: 0
  - Duration of awakening: 0 min
  - Final time you woke up: 08:30
  - Time you got up from bed: 08:35
  - Duration of total sleep: 8 hours 35 min

- **Night 12**
    - Time you went to bed: 21:00
    - Time you tried to fall asleep: 21:20
    - Amount of time it took you to fall asleep: 20 min
    - # of awakenings at night: 1
    - Duration of awakening: 25 min
    - Final time you woke up: 07:28
    - Time you got up from bed: 07:30
    - Duration of total sleep: 9 hours 23 min
- **Night 13**
    - Time you went to bed: 23:00
    - Time you tried to fall asleep: 23:00
    - Amount of time it took you to fall asleep: 10 min
    - # of awakenings at night: 1
    - Duration of awakening: 1 min
    - Final time you woke up: 07:00
    - Time you got up from bed: 07:05
    - Duration of total sleep: 7 hours 49 min
- **Night 14**
    - Time you went to bed: 22:10
    - Time you tried to fall asleep: 23:50
    - Amount of time it took you to fall asleep: 2 min
    - # of awakenings at night: 0

    - Duration of awakening: 30 min
    - Final time you woke up: 05:20
    - Time you got up from bed: 05:30
    - Duration of total sleep: 4 hours 58 min

Nightly Activity

This section shows the averages of your self-recorded 2-week sleep activity data.

- Avg. time you went to bed: 22:22
    - On average, you went to bed at 10:22pm.
- Avg. time you fell asleep: 22:38
    - On average, you fell asleep at 10:38pm.
- Avg. amount of time it took you to fall asleep: 7 min 0 sec
    - On average, it took you 7 min 0 sec to try to fall asleep.
- Avg. # of awakenings at night: 0.69
    - On average, you woke up 0.69 times during the night.
- Avg. duration of awakenings: 8 min 0 sec
    - On average, you were awake for 8 min 0 sec during the night.
- Avg. time you woke up: 06:44
    - On average, you woke up at 6:44am.
- Avg. time you got up from bed: 06:49
    - On average, you got out of bed at 6:49am.
- Avg. duration of total nightly sleep: 7 hr 50 min
    - On average, you slept for 7 hr 50 min each night.

Your Sleep Efficiency

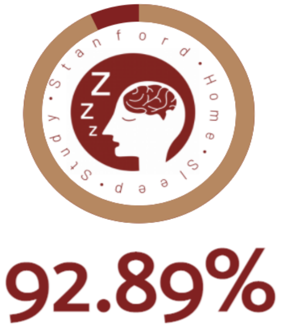

Sleep efficiency is calculated by dividing the amount of time spent asleep (in minutes) by the total amount of time in bed (in minutes).

Sleep Buddy Activity

This section shows how many total teeth grinding/clenching events were recorded each night based on jaw muscle activity.

Key:

- * means data likely incomplete/only a few hours recorded
- - - means no data for that night

	Total Grinding Events Recorded
Night 1	144
Night 2	352
Night 3	670
Night 4	45
Night 5	190
Night 6	72
Night 7	89
Night 8	312
Night 9	56

Night 10	46
Night 11	67
Night 12	232
Night 13	783
Night 14	111
Total Nights of Data	14

Note: there may be certain nights that show missing data, marked above with dashes. These are the nights when the Sleep Buddy may not have been set correctly and was unable to begin data collection. Having a few missing nights of Sleep Buddy data is not uncommon and does not exclude us from using your data.

Actigraphy Report

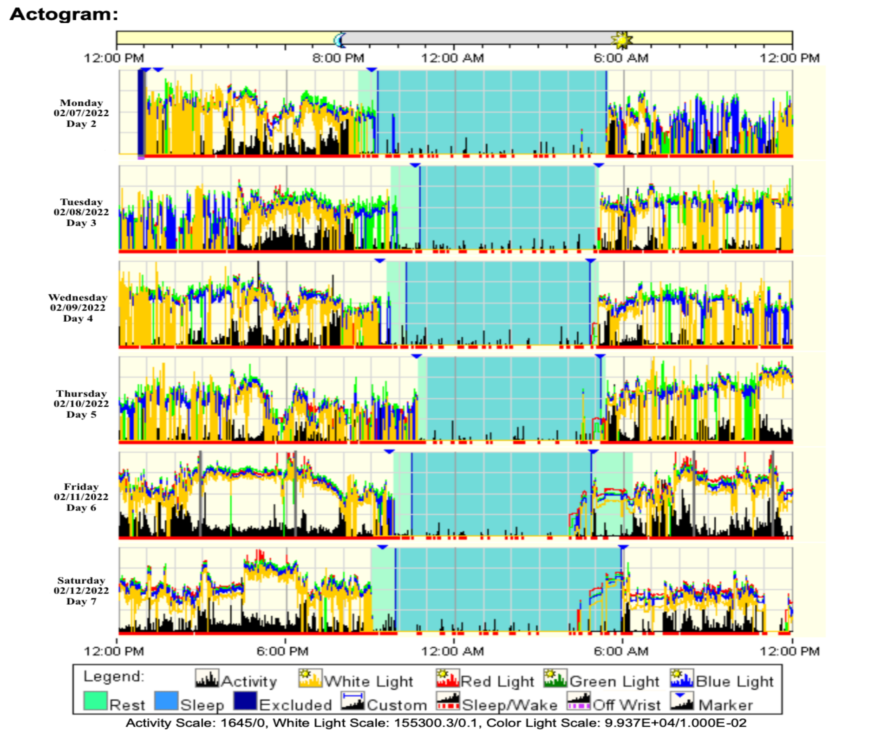

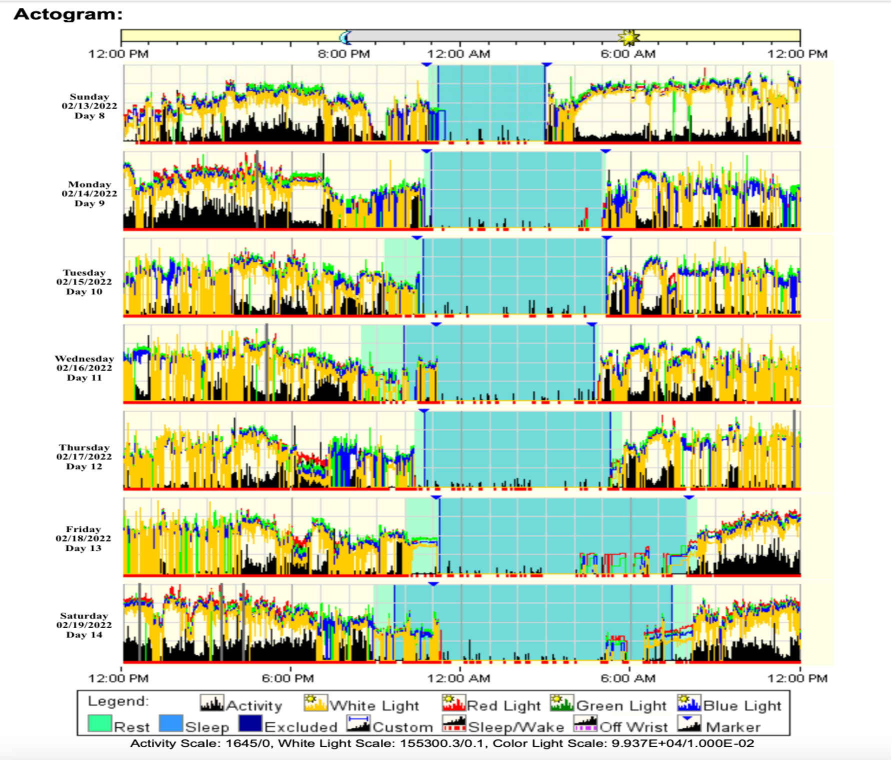

Actogram:

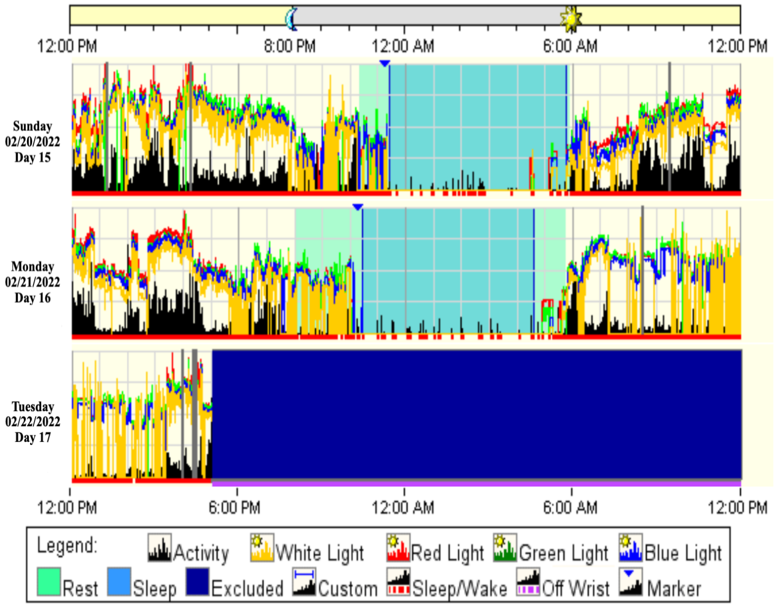

Activity Scale: 1645/0, White Light Scale: 155300.3/0.1, Color Light Scale: 9.937E+04/1.000E-02

Personality Results: Big 5

This section shows your results from the personality survey.

- Extraversion:
  - As an extraverted individual, you tend to be energized by being with and connecting with people. It is a joy for you to strike up conversation, it is easy for you to be emotionally expressive with others. You are naturally energetic, talkative, and not afraid of asserting yourself in your work or social life and tend to shine in social settings.
- Agreeableness:
  - Tending to be naturally trusting and forgiving, you are compassionate and encouraging to those that surround you. You are an excellent collaborator and thrive when you work in groups and experience a great deal of empathy when interacting with others.
- Conscientiousness:
  - You are self-disciplined, thorough, and highly organized when it comes to your work and personal endeavors. The people in your life might describe you as reliable and prompt, possessing a high attention to detail towards each task you choose to take on.
- Neuroticism:
  - Generally, you are emotionally resilient, confident, and are skilled in dealing with stress in a healthy way, possessing a strong ability to bring yourself into a relaxed headspace when you need to.
- Openness:

  - You enjoy playing with and discussing new ideas, and are excited to think in more abstract ways. As a naturally creative, adventurous, and insightful person, you may have a wide variety of interests and are generally unafraid of diving into unfamiliar activities and seeking out new life experiences.

Personality Results: Emotional Regulation

This section shows your results and analysis from the personality survey.

- Emotional Regulation Capacity: Cognitive Reappraisal:
  - You are highly capable of managing your emotional experiences. Regardless of whether you attempt this often, you possess the ability to shift the way you are thinking about a given situation so that you can feel more positive emotions (such as joy or amusement).
- Emotional Regulation Capacity: Expressive Suppression:
  - When you really want to, you are very careful expressing an emotion (i.e., anger or excitement) when you are feeling it.
- Emotional Regulation Frequency: Cognitive Reappraisal:
  - Frequently, when faced with a stressful situation, you tend to change what you are thinking about such that you can feel a stronger positive emotion (such as amusement or joy).
- Emotional Regulation Frequency: Expressive Suppression:
  - As an expressive and sensitive individual, you are not afraid to present the way you're feeling in the moment, especially in response to an exciting or difficult experience, rather than attempt to suppress it. relationships with a delicate and thoughtful approach.
