# Supplementary material for: The Role of Emotion Regulation, Affect, and Sleep in Individuals With Sleep Bruxism and Those Without: Protocol for a Remote Longitudinal Observational Study
Source: JMIR Res Protoc. 2023 Aug 24;12:e41719. doi: 10.2196/41719 (PMC10485716; doi:10.2196/41719)
Supplement: Multimedia Appendix 6 [file resprot_v12i1e41719_app6.pdf]

Multimedia Appendix 6. Baseline (T1) Individual-Difference Assessment

1. Demographic Information 2

2. Sleep Bruxism Questionnaire 2

3. Oral Health Impact Profile 14 (OHIP-14) 5

4. Patient-Reported Outcomes Measurement Information System (PROMIS) Pain Intensity – Short Form 3a V1.0 Modified for Orofacial Pain 5

5. Emotion Regulation Questionnaires 6

6. Affect Questionnaires 8

7. Pittsburgh Sleep Quality Index (PSQI) 9

8. Attention Check Items in Baseline (T1) Individual Differences Assessment 9

References 11

The Baseline (T1) Individual Differences Assessment contains the following: (1) Demographic Information survey, (2) Sleep Bruxism Questionnaire, (3) Oral Health Impact Profile 14 (OHIP-14), (4) Patient-Reported Outcomes Measurement Information System (PROMIS) Pain Intensity Short Form Modified for Orofacial Pain, (5) Emotion Regulation Questionnaires, (6) Affect Questionnaires, (7) Pittsburgh Sleep Quality Index, and (8) Attention Check Items in Baseline (T1) Individual Differences Assessment. Research assistants emailed

participants an online link to complete the individual differences assessment and EMA training before the web-based introduction session. Automated emails sent by REDCap reminded participants of the requirement to complete the individual differences assessment and EMA training before their first study session.

1. Demographic Information

In the web-based individual differences assessment, "___" seen below is replaced by the actual value that the participant reported in the web-based prescreen survey.

1. Please confirm your sex is ___.
2. Please confirm your age is ___.
3. Please confirm your ethnicity is ___.
4. Please confirm your race is ___.
5. Please confirm your marital status is ___.
6. What is the highest grade (or year) of regular school you have completed?
7. Please confirm the highest degree you have earned is ___.
8. Please confirm your current main daily activities and/or responsibilities: ___
9. Please confirm your main occupation or job: ___
10. Please confirm your approximate total family income per YEAR: ___
11. How many people are currently living in your household, including yourself?

2. Sleep Bruxism Questionnaire

The Sleep Bruxism Questionnaire (SBQ) consists of the following instructions and items:

Please answer the following questions.

1. Do you grind your teeth during sleep?
2. Do you have any cracked teeth?
3. Are your teeth worn down or have they become shorter over time?
4. Are your teeth sensitive to hot, cold, or acidic foods?
5. Has the texture of your teeth surface changed over time?

Over the past 6 months, how often have you experienced any of the following in the morning? If the frequency varies, please choose the higher option.

6. Over the past 6 months, how often have you experienced headache in the morning? If the frequency varies, please choose the higher option.
7. Over the past 6 months, how often have you experienced jaw soreness or ache in the morning? If the frequency varies, please choose the higher option.
8. Over the past 6 months, how often have you experienced toothache in the morning? If the frequency varies, please choose the higher option.
9. Over the past 6 months, how often have you experienced earache in the morning? If the frequency varies, please choose the higher option.
10. Over the past 6 months, how often have you experienced sore neck in the morning? If the frequency varies, please choose the higher option.
11. Over the past 6 months, how often have you experienced fatigue in your jaw in the morning? If the frequency varies, please choose the higher option.
12. Over the past 6 months, how often have you experienced jaw lock the morning? If the frequency varies, please choose the higher option.
13. Over the past 6 months, how often have you experienced clicking jaw in the morning? If the frequency varies, please choose the higher option.

14. Over the past 6 months, how often have you experienced tension or stiffness in your jaw in the morning? If the frequency varies, please choose the higher option.

15. Over the past 6 months, how often have you experienced tension or fatigue of your neck in the morning? If the frequency varies, please choose the higher option.

Over the past 6 months, how often have you experienced the following during the night? If the frequency varies, please choose the higher option.

16. Over the past 6 months, how often have you been woken up during the night by your teeth grinding? If the frequency varies, please choose the higher option.

17. Over the past 6 months, how often have you been not able to fall asleep again after being woken up by your teeth grinding during the night? If the frequency varies, please choose the higher option.

18. Over the past 6 months, how often have you been woken up by your partner due to your teeth grinding during the night? If the frequency varies, please choose the higher option.

19. Over the past 6 months, how often have you been having nightmares while grinding your teeth during the night? If the frequency varies, please choose the higher option.

20. Over the past 6 months, how often have you been not able to sleep because of a sore jaw, toothache, or headache due to your teeth grinding during the night? If the frequency varies, please choose the higher option.

3. Oral Health Impact Profile 14 (OHIP-14)

The Oral Health Impact Profile [1] assesses patients' perception of the impact of oral disorders on their quality of life. Whereas the original survey does not specify the time period, we modified the survey to ask about the past year. The scale includes questions such as, “Have you had trouble pronouncing any words because of problems with your teeth, mouth, or dentures?”. The 14 questions have answer options on a 5-point Likert-type frequency scale ranging from “very often” to “never.”

4. Patient-Reported Outcomes Measurement Information System (PROMIS) Pain Intensity – Short Form 3a V1.0 Modified for Orofacial Pain

The Patient-Reported Outcomes Measurement Information System (PROMIS) Pain Intensity – Short Form 3a V1.0 [2] measures the respondent’s subjective pain in the past 7 days and at present. We adapted the Pain Intensity Short Form to ask about orofacial pain:

In the following questions, please refer to your experience in the past 7 days. The following questions refer to any pain you might have experienced localized to the region above the neck, in front of the ears and below the eye-ear plane, as well as pain within the oral cavity. Please respond to each item by marking one box per row. Answer options lie on a 5-point Likert-type scale ranging from “Had no pain” to “Very severe.”

1. How intense was your pain at its worst?
2. How intense was your average pain?
3. What is your level of pain right now?

3. What is your level of pain right now?

5. Emotion Regulation Questionnaires

1. Emotion Regulation Questionnaire Frequency (ERQ-F) [3]

In this questionnaire, participants respond with respect to a general timeframe. Each ER strategy is assessed with 5 items. Answers are obtained on a 7-point Likert-type scale, ranging from “strongly disagree” (1) to “strongly agree” (7).

2. Emotion Regulation Questionnaire Distraction Frequency (ERQ-DF):

This questionnaire consists of 4 items answered on a 7-point Likert scale, ranging from “strongly disagree” (1) to “strongly agree” (7). Participants respond with respect to a general timeframe.

We would like to ask you some questions about your emotional life, in particular, how you control (that is, regulate and manage) your emotions. The questions below address your emotional life, specifically what you think about or what you pay attention to. Although some of the following questions may seem similar to one another, they differ in important ways.

1. When I'm faced with a stressful situation, I make myself think about something else to help me stay calm.

2. When I want to feel more positive emotion, I think about something more positive than the situation I'm in.

3. I control my emotions by changing what I pay attention to.

4. When I want to feel less negative emotion, I think about something different from the situation I'm in.

3. Emotion Regulation Questionnaire Self Efficacy (ERQ-SE) [3]

Answers were obtained on a 7-point Likert-type scale, ranging from "strongly disagree" (1) to "strongly agree" (7) with respect to a general timeframe.

4. Emotion Regulation Questionnaire Distraction Self Efficacy (ERQ-DSE)

Answers were obtained on a 7-point Likert-type scale, ranging from "strongly disagree" (1) to "strongly agree" (7) with respect to a general timeframe:

We would now like to ask you some questions about HOW CAPABLE you are of controlling (meaning, regulating and managing) your attention to emotional events.

Note that a person might almost never control their attention to emotion in everyday life, and yet might be very capable of doing so if he or she really wanted to. Conversely, someone might frequently try to control their attention to emotion in everyday life, but not feel very capable.

Although some of the following questions may seem similar to one another, they differ in important ways.

1. When I really want to, I am very capable of making myself think about something else during a stressful situation in order to stay calm.
2. When I really want to, I am very capable of thinking about something more positive than the situation I'm in when I want to feel more positive emotion.
3. When I really want to, I am very capable of controlling my emotions by changing what I pay attention to.
4. When I really want to, I am very capable of thinking about something different from the situation I'm in when I want to feel less negative emotion.

6. Affect Questionnaires

1. Patient-Reported Outcomes Measurement Information System (PROMIS) Depression Short Form 8a – v1.0 (PROMIS Depression Scale) [4]

This questionnaire includes 8 statements related to depressed mood in the last 7 days on a 5-point Likert-type frequency scale ranging from 1 "never" to 5 "always." It includes statements such as "In the last 7 days, I felt hopeless" and "In the last 7 days, I felt I had nothing to look forward to." Greater scores indicate greater depression.

2. Patient-Reported Outcomes Measurement Information System (PROMIS) Anxiety 8a – Short Form v1.0 (PROMIS Anxiety Scale) [4]

This questionnaire includes 8 statements on a 5-point Likert-type frequency scale ranging from 1 "never" to 5 "always." It includes statements such as "In the last 7 days, I felt fearful" and "In the last 7 days, I found it hard to focus on anything other than my anxiety." Greater scores indicate greater anxiety.

3. Positive and Negative Affect Schedule (PANAS) [5]

The original survey uses multiple timeframes, including in the moment, today, the past few days, the past week, the past few weeks, the past year, and lifetime, whereas this modified survey asks how participants felt in the past 2 weeks. This questionnaire asks how participants generally felt on a 5-point Likert-type frequency scale ranging from 1 "very slightly or not at all" to 5 "extremely." It contains 20 items, 10 relating to positive affect (eg, "interested," "excited," or "strong") and 10 relating to negative affect (eg, "distressed," "upset," or "guilty").

4. Perceived Stress Scale (PSS) [6]

This scale includes 10 questions related to perceived stress in the last month on a 5-point Likert-type frequency scale ranging from 1 "never" to 5 "very often." It includes questions such

as “In the last month, how often have you been upset because of something that happened unexpectedly?” and “In the last month, how often have you felt confident about your ability to handle your personal problems?” The overall score is derived by reverse scoring the 4 positively stated items and then adding up all 10 scores.

7. Pittsburgh Sleep Quality Index (PSQI)

The Pittsburgh Sleep Quality Index (PSQI) [7] is a questionnaire that includes 9 questions relating to sleep habits in the last month. Various combinations of questions form subsections, which include: subjective sleep quality, sleep latency, sleep duration, habitual sleep efficiency, sleep disturbances, use of sleeping medications, and daytime dysfunction. Each subsection is weighed equally and graded on a 0 to 3 scale. The 7 combined scores form one composite score on a scale of 0 to 21 that represents sleep quality; the higher the score, the poorer the sleep quality. Example questions include “During the past month, how often have you had trouble staying awake while driving, eating meals, or engaging in social activity?” and “During the past month, how much of a problem has it been for you to keep up enthusiasm to get things done?”

8. Attention Check Items in Baseline (T1) Individual Differences Assessment

Inclusion criteria for participation in the study requires that potential participants pass 8 of a total of 9 attention checks in the T1 individual differences assessment [8].

1. In the past 7 days, could you please skip this question, so that we know the data are being collected appropriately?
2. When you read this, could you please select "sometimes" in order to verify that the browser works properly and that we are collecting all the responses?

3. If you are reading this, please choose "somewhat of a problem" to confirm that you are paying attention to the questions.
4. Have you read this question? If yes, please choose the "occasionally" option so that we know the data are being collected successfully.
5. In order to verify that the web browser you are using works properly and that we are collecting all your answers, please select disagree strongly
6. I feel as if you read this message please choose 1. 1 = completely disagree ; 5 = completely agree
7. When you read this question, please choose "sometimes" to show us that you are actually paying attention.
8. Please choose “false" so we know that you are reading this.

Please choose "very unlike me" and "very like me" to confirm that you are reading this.

References

1. Slade GD. Derivation and validation of a short-form oral health impact profile. Community Dent Oral Epidemiol 1997 Aug;25(4):284-290 [doi: 10.1111/j.1600-0528.1997.tb00941.x] [Medline: 9332805]
2. Cella D, Riley W, Stone A, Rothrock N, Reeve B, Yount S, PROMIS Cooperative Group. The Patient-Reported Outcomes Measurement Information System (PROMIS) developed and tested its first wave of adult self-reported health outcome item banks: 2005-2008. J Clin Epidemiol 2010 Nov;63(11):1179-1194 [FREE Full text] [doi: 10.1016/j.jclinepi.2010.04.011] [Medline: 20685078]
3. Gross JJ, John OP. Individual differences in two emotion regulation processes: implications for affect, relationships, and well-being. J Pers Soc Psychol 2003 Aug;85(2):348-362 [doi: 10.1037/0022-3514.85.2.348] [Medline: 12916575]
4. Pilkonis PA, Choi SW, Reise SP, Stover AM, Riley WT, Cella D, PROMIS Cooperative Group. Item banks for measuring emotional distress from the Patient-Reported Outcomes Measurement Information System (PROMIS®): depression, anxiety, and anger. Assessment 2011 Sep;18(3):263-283 [FREE Full text] [doi: 10.1177/1073191111411667] [Medline: 21697139]
5. Watson D, Clark LA, Tellegen A. Development and validation of brief measures of positive and negative affect: the PANAS scales. J Pers Soc Psychol 1988 Jun;54(6):1063-1070 [doi: 10.1037//0022-3514.54.6.1063] [Medline: 3397865]
6. Cohen S, Kamarck T, Mermelstein R. A global measure of perceived stress. J Health Soc Behav 1983 Dec;24(4):385-396 [FREE Full text] [doi: 10.2307/2136404]

7. Buysse DJ, Reynolds 3rd CF, Monk TH, Berman SR, Kupfer DJ. The Pittsburgh sleep quality index: a new instrument for psychiatric practice and research. Psychiatry Res 1989 May;28(2):193-213 [doi: 10.1016/0165-1781(89)90047-4] [Medline: 2748771]
8. Berinsky AJ, Margolis MF, Sances MW. Separating the shirkers from the workers? Making sure respondents pay attention on self-administered surveys. Am J Pol Sci 2014 Jul;58(3):739-753 [doi: 10.1111/ajps.12081]
