# Supplementary material for: The Role of Emotion Regulation, Affect, and Sleep in Individuals With Sleep Bruxism and Those Without: Protocol for a Remote Longitudinal Observational Study
Source: JMIR Res Protoc. 2023 Aug 24;12:e41719. doi: 10.2196/41719 (PMC10485716; doi:10.2196/41719)
Supplement: Multimedia Appendix 7 [file resprot_v12i1e41719_app7.pdf]

Multimedia Appendix 7. Poststudy (T2) Individual-Difference Assessment

1. Questionnaires 1

2. In-House Attention Checks in Poststudy (T2) Individual Differences Assessment 2

References 3

1. Questionnaires

Upon the end of the 14-day ambulatory assessment, participants received an online link via email to complete a poststudy (T2) individual differences assessment with questionnaires about affect and sleep. The T2 Individual Differences Assessment contains questionnaires about affect and sleep, listed below.

Questionnaires pertaining to affect:

1. Patient-Reported Outcomes Measurement Information System (PROMIS) Depression Short Form 8a – v1.0 (PROMIS Depression Scale) [1]
2. Patient-Reported Outcomes Measurement Information System (PROMIS) Anxiety 8a – Short Form v1.0 (PROMIS Anxiety Scale) [1]
3. Positive and Negative Affect Schedule (PANAS) [2]
4. Perceived Stress Scale (PSS) [3]

Questionnaires pertaining to sleep:

1. Pittsburgh Sleep Quality Index (PSQI) [4]

2. In-House Attention Checks in Poststudy (T2) Individual Differences Assessment

Inclusion criteria for participation in the study required that potential participants pass 8 of a total of 9 attention checks in the T1 individual differences assessment [5]. The questions are seen below.

1. During the past month if you are reading this, please choose "somewhat of a problem" to confirm that you are paying attention to the questions.
2. When you read this, could you please select "sometimes" in order to verify that the browser works properly and that we are collecting all the responses?
3. During the past month if you are reading this, please choose "somewhat of a problem" to confirm that you are paying attention to the questions.
4. I can learn, please enter slightly disagree below.
5. If I want to, please enter “strongly agree” below.
6. I feel like, please select number 2.

References

1. Pilkonis PA, Choi SW, Reise SP, Stover AM, Riley WT, Cella D, PROMIS Cooperative Group. Item banks for measuring emotional distress from the Patient-Reported Outcomes Measurement Information System (PROMIS®): depression, anxiety, and anger. Assessment 2011 Sep;18(3):263-283 [FREE Full text] [doi: 10.1177/1073191111411667] [Medline: 21697139]
2. Watson D, Clark LA, Tellegen A. Development and validation of brief measures of positive and negative affect: the PANAS scales. J Pers Soc Psychol 1988 Jun;54(6):1063-1070 [doi: 10.1037//0022-3514.54.6.1063] [Medline: 3397865]
3. Cohen S, Kamarck T, Mermelstein R. A global measure of perceived stress. J Health Soc Behav 1983 Dec;24(4):385-396 [FREE Full text] [doi: 10.2307/2136404]
4. Buysse DJ, Reynolds 3rd CF, Monk TH, Berman SR, Kupfer DJ. The Pittsburgh sleep quality index: a new instrument for psychiatric practice and research. Psychiatry Res 1989 May;28(2):193-213 [doi: 10.1016/0165-1781(89)90047-4] [Medline: 2748771]
5. Berinsky AJ, Margolis MF, Sances MW. Separating the shirkers from the workers? Making sure respondents pay attention on self-administered surveys. Am J Pol Sci 2014 Jul;58(3):739-753 [doi: 10.1111/ajps.12081]
