# Supplementary material for: The Role of Emotion Regulation, Affect, and Sleep in Individuals With Sleep Bruxism and Those Without: Protocol for a Remote Longitudinal Observational Study
Source: JMIR Res Protoc. 2023 Aug 24;12:e41719. doi: 10.2196/41719 (PMC10485716; doi:10.2196/41719)
Supplement: Multimedia Appendix 8 [file resprot_v12i1e41719_app8.pdf]

Multimedia Appendix 8. National Institutes of Health peer-review report

SUMMARY STATEMENT

(Privileged Communication)

PROGRAM CONTACT:
[redacted]

Release Date: **11/09/2016**
Revised Date:

Application Number: **1 R01 DE026771-01**

Principal Investigator

GROSS, JAMES J

Applicant Organization: STANFORD UNIVERSITY

Review Group: **MESH**
Biobehavioral Mechanisms of Emotion, Stress and Health Study Section

Meeting Date: **10/24/2016**
Council: **JAN 2017**
Requested Start: **04/01/2017**

RFA/PA: **PA16-160**
PCC: **Q2A**

Project Title: **Emotion Dysregulation and Sleep-Time Masticatory Muscle Activity in Sleep Bruxism**
SRG Action: **Impact Score:18 Percentile:7**
Next Steps: **Visit http://grants.nih.gov/grants/next_steps.htm**
Human Subjects: **30-Human subjects involved - Certified, no SRG concerns**
Animal Subjects: **10-No live vertebrate animals involved for competing appl.**
Gender: **1A-Both genders, scientifically acceptable**
Minority: **1A-Minorities and non-minorities, scientifically acceptable**
Children: **3A-No children included, scientifically acceptable**
Clinical Research - not NIH-defined Phase III Trial

Project Year	Direct Costs Requested	Estimated Total Cost
1	[redacted]	[redacted]
2		
3		
4		
5		
TOTAL	[redacted]	[redacted]

ADMINISTRATIVE BUDGET NOTE: The budget shown is the requested budget and has not been adjusted to reflect any recommendations made by reviewers. If an award is planned, the costs will be calculated by Institute grants management staff based on the recommendations outlined below in the COMMITTEE BUDGET RECOMMENDATIONS section.

1R01DE026771-01 GROSS, JAMES

RESUME AND SUMMARY OF DISCUSSION: This study will use experimental manipulation of emotion regulation (ER) to test a neurobiological model linking deficits in ER to sleep bruxism (SB). Sleep bruxism is a common problem associated with masticatory muscle activity (MMA) that leads to sleep disturbance, pain, and joint degeneration; ER could provide a modifiable biobehavioral target for reducing SB and thereby improving symptoms and quality of life in those with SB. Although the premise—that deficits in ER lead to increased amygdala activation persistent during sleep, resulting in increased MMA—was generally viewed as strong, during discussion panel members agreed that a minor limitation of the application is the insufficient consideration of alternative explanations and of the potential for bidirectional relationships between these variables. Numerous strengths of the project were discussed, including its innovative and clinically significant model that considers the contribution of the amygdala and prefrontal cortex, an eminent research team, preliminary data that suggest feasibility and support study hypotheses, state of the art imaging procedures, and exemplary rigorous methodology. Minor concerns did not detract from the overall assessment that this is a highly significant project that will further our understanding of putative biobehavioral pathways to a common sleep problem.

DESCRIPTION (provided by applicant): The overall goal of this proposal is to examine the role of emotion regulation (ER) in sleep bruxism (SB). SB is characterized by extreme levels of masticatory muscle activity (MMA) during sleep, expressed as teeth grinding or clenching. SB may proximally lead to tooth damage, orofacial pain, and impaired sleep; predispose to the development of joint degenerative disorders; and ultimately present a preclinical sign for a broader range of neurodegenerative disorders. However, there is as yet no curative treatment for SB, and the mechanisms underlying SB are not well understood. To fill this gap, we propose an integrative neurobiological framework that focuses on the involvement of impaired downregulation of wake-time emotion in SB. We will test basic tenets of this framework by addressing four major aims: Aim 1 tests differences in task-related neural activation during ER in individuals with SB (SB+) vs. matched controls (SB−). Aim 2 investigates direct and indirect pathways associating task- related prefrontal cortex activation during ER with MMA during sleep among SB+ and SB−. Aim 3 examines whether neural activation during ER can be experimentally manipulated in SB+. Aim 4 addresses causal mechanisms by investigating whether more efficient task-related prefrontal cortex activation during ER decreases MMA during sleep in SB+ through decreased task-related emotional activation of the amygdala. 100 SB+ and 50 SB− will be defined based on polysomnographic research diagnostic criteria. Functional activations of brain regions of interest during an ER task will be assessed and ambulatory monitoring of MMA during sleep will be conducted. In SB+, ER will be experimentally manipulated and effects will be assessed on functional activations of brain regions of interest during the ER task as well as on MMA during sleep. The proposed work is part of a programmatic translational research agenda to develop novel effective therapies that target ER processes to alleviate affective, sleep, and stress-induced neurodegenerative disorders.

PUBLIC HEALTH RELEVANCE: The goal of this proposal is to elucidate whether in sleep bruxism difficulties regulating wake-time emotional responses lead to heightened masticatory muscle activity during sleep through heightened levels of emotional activation of the amygdala that continue into sleep-time. The long-term objective of this research is to understand whether emotion dysregulation represents a core feature of sleep bruxism that may be targeted in the development of psychosocial treatments for this and other disorders.

CRITIQUE 1:

Significance: 3
Investigator(s): 2

Innovation: 2
Approach: 2
Environment: 1

Overall Impact: This high impact and significant project examines whether disturbances in emotion regulation (ER) contribute to sleep bruxism (SB), a common condition characterized by masticatory muscle activity (MMA) that leads to orofacial pain, sleep disturbance, and joint degeneration. The application hypothesizes that deficits in ER during the day lead to heightened amygdala activation that persists during sleep, resulting in increased MMA in SB. An integrative neurobiological framework is proposed that builds on a strong scientific premise and the experiments are methodologically rigorous. A well-validated ER task during fMRI will be used to examine the role of the PFC and amygdala in impaired ER and in the generation of MMA evaluated during sleep in individuals with and without SB. SB is determined by polysomnographic screening/clinical diagnosis. The study will also examine whether an experimental cognitive reappraisal manipulation can improve ER in sleep bruxism, and whether improved ER leads to decreases in MMA during sleep. Preliminary evidence indicates that SB is associated with a failure to recover from negative emotion, impaired ER along with PFC hypoactivation and amygdala hyperactivation during the fMRI ER task. The application also provides cross-sectional evidence that negative affect mediates the effects of impaired reappraisal capacity on SB symptom exacerbation, and evidence that cognitive reappraisal can improve ER in SB. Four aims use the ER task in fMRI studies to evaluate whether task-related neural activation differs between individuals with SB and controls, whether experimental manipulation can improve ER in SB, and whether improved ER leads to decreases in MMA during sleep. Strengths include the rigorous research design, the power and statistical analysis plans, inclusion/exclusion/recruitment plans, and examination of sex as a biological variable. Another strength is the use of an innovative multi-method approach to test the model. The outstanding investigative team and environment enhance the feasibility of the project. In terms of limitations, the project does not fully discuss alternative hypotheses and possible bidirectional pathways, (i.e. that MMA might lead to sleep disruption, which in turn increases wake time negative affect and poor ER, and that this persists during sleep to further increase MMA). The clinical significance of this project depends on whether the reappraisal manipulation is able to reduce SB symptoms and whether this effect is mediated by a reduction in negative emotion. Therefore, a preliminary longitudinal study demonstrating these relations using the self-report measures in PS6 would bolster the scientific premise and rationale for the more labor intensive fMRI and sleep studies. Overall, this is an outstanding project. The knowledge gained from these studies could lead to the development of new treatments that target ER in SB and other stress-related conditions.

1. Significance:

Strengths

- Sleep bruxism (SB) is a prevalent condition that leads to tooth damage, orofacial pain, TMJ, headache, tinnitus sleep disturbance, and joint degeneration. Understanding whether ER contributes to the induction and maintenance of SB might lead to the development of new treatments.
- Current SB treatments focus on symptom management and there is no cure. Although SB is believed to be centrally generated, management strategies focus on peripheral symptom management rather than targeting central mechanisms. If this central hypothesis is supported, it could lead to new and more effective psychological interventions.
- The results of this study might have implications for understanding the role ER in other affective, sleep, and neurodegenerative disorders.

Weaknesses

- The preliminary results section includes a cross-sectional analysis (PS 6) of the relations among ER, SB symptoms, and general negative affect, which suggests that the effects of impaired reappraisal capacity on SB symptom exacerbation are mediated by negative affect. This is followed by another study (PS 7) showing that negative affect is reduced in SB following an experimental manipulation of ER. Although these findings are consistent with the central hypothesis, the model and the efficacy of the reappraisal manipulation in reducing negative affect and SB symptoms has not been verified in a longitudinal study prior to the proposed work. This is important because the clinical significance of the fMRI studies depends on the efficacy of the reappraisal manipulation in reducing SB symptoms and on the validity of the underlying mediation model.
- The project does not fully discuss alternative hypotheses and bidirectional pathways, (i.e., that MMA might lead to sleep disruption, which in turn increases wake time negative affect and poor ER, and that this persists during sleep to further increase MMA).

2. Investigator(s):

Strengths

- This is an outstanding investigative team. The Principal Investigator is a clinical psychologist with expertise in affect regulation. Dr. Kushida, Co-Investigator, is medical director of the Stanford University Sleep Medicine Center. Dr. Manber, Co-Investigator, is director of the Stanford University Sleep Enhancement Program with expertise in longitudinal sleep research.
- Dr. Lavigne, expertise in SB and orofacial pain disorder, is serving as an external consultant.

Weaknesses

- Although the Principal Investigator is an expert in emotion regulation, he is not an expert in sleep bruxism or sleep. That said, the expertise of the investigative team, and their prior history of collaboration, make this a minor concern.

3. Innovation:

Strengths

- The multi-method approach will use fMRI to measure the central mechanisms underlying ER, the effects of experimental manipulation of ER on neural activation, and the consequences of this manipulation on ambulatory night time MMA. This approach is highly innovative and might determine the causal mechanisms.

Weaknesses

- Although the combination of methods provide an innovative approach to test the central hypothesis, the individual methods are not especially innovative; this is an very minor concern.

4. Approach:

Strengths

- Preliminary questionnaire data support the feasibility of recruiting sufficient numbers of SB and HC participants.
- Additional preliminary data provides support for the central hypothesis, demonstrating that SB is associated with prefrontal cortex (PFC) hypoactivation and amygdala hyperactivation during an fMRI ER task. Other data shows that SB is associated with a failure to recover from negative

emotion and impaired ER during tasks, and impaired ER and negative affect in daily life. Importantly, an experimental manipulation (reappraisal) was shown to improve ER in SB.

- Dr. Kushida will conduct prescreening polysomnography (PSG) to determine presence or absence of SB and initial clinical/dental/TMD screening.
- Neural activation during emotion generation and ER is measured using fMRI, MMA is measured through ambulatory monitoring during sleep, and ER is experimentally manipulated to test causality.
- Four logical aims test the assumptions and causal role of impaired ER in the generation of MMA in their integrative model of SB. Aim 1 evaluates whether task-related neural activation in PFC and amygdala during an ER task is impaired in SB. Aim 2 tests whether impaired task-related PFC activation leads to heightened masticatory muscle activity (MMA) during sleep through increased amygdala activation. Aim 3 will test whether an experimental manipulation, reappraisal, can improve task-related neural activation in PFC during ER in SB. Aim 4 will determine whether improved ER leads to decreases in MMA during sleep.
- Rigor and reproducibility are enhanced by the critical design decision section, the use of a well-validated multimethod approach, detailed inclusion/exclusion criteria and recruitment strategy, power and statistical analysis plan.
- The project will be registered on the Open Science Framework (osf.io) to increase the transparency and reproducibility of this research.
- Both genders are included. Menstrual phase, hormonal contraceptives, and menopausal status will be assessed allowing examination of sex as a biological variable.

Weaknesses

- None noted.

5. Environment:

Strengths

- The environment and resources at Stanford are outstanding, including the Stanford Center for Cognitive and Neurobiological Imaging (Psychology), Stanford Psychophysiology Laboratory (Gross), and the Stanford Sleep Medicine Center (Kushida).

Weaknesses

- No concerns.

Protections for Human Subjects:

Acceptable Risks and/or Adequate Protections

- The project adequately addressed concerns regarding risks.

Data and Safety Monitoring Plan (Applicable for Clinical Trials Only):

Not Applicable (No Clinical Trials)

Inclusion of Women, Minorities and Children:

- Sex/Gender: Distribution justified scientifically
- Race/Ethnicity: Distribution justified scientifically
- For NIH-Defined Phase III trials, Plans for valid design and analysis: Not applicable

- Inclusion/Exclusion of Children under 18: Excluding ages <18; justified scientifically
- Appropriate rationales are provided, both genders, plan to recruit diverse sample.

Vertebrate Animals:
Not Applicable (No Vertebrate Animals)

Biohazards:
Not Applicable (No Biohazards)

Authentication of Key Biological and/or Chemical Resources:
Not Applicable (No Relevant Resources)

Budget and Period of Support:
Recommend as Requested

CRITIQUE 2:

Significance: 2
Investigator(s): 1
Innovation: 3
Approach: 3
Environment: 1

Overall Impact: This study proposes to examine associations among emotion regulation and symptoms of bruxism during sleep in adults with and without a clinical diagnosis of sleep bruxism (SB+ and SB). The project is based on the novel neurobiological hypothesis (scientific premise) that disrupted emotional regulation during waking contributes to increased masticatory muscle activity during sleep via decreased cognitive control during sleep and persisting activation of the amygdala. A series of four integrated studies are proposed to examine components of the proposed model. Strengths of the application include its conceptual underpinnings, innovative and clinically significant model, and world-class research team. The applications is also very well-written.

1. Significance:

Strengths

- Sleep bruxism is common and is associated with adverse consequences to dental health, pain and quality of life.
- Emotion regulation might provide a modifiable biobehavioral pathway for reducing sleep bruxism (SB) and improving dental health and quality of life in SB+ individuals.

Weaknesses

- None noted.

2. Investigator(s):

Strengths

- Investigators are highly productive scientists with the requisite expertise to conduct the proposed study. The Principal Investigator, James Gross, is a leading expert in the neurobiological bases of emotion regulation. Co-Investigators, Clete Kushida and Rachel Manber are leading experts in sleep medicine with complementary expertise in medical and behavioral sleep disorders. Consultant Gilles Lavigne has a productive clinical research program focused on the causes and treatment of sleep bruxism.

Weaknesses

- None noted.

3. Innovation:

Strengths

- The proposed neurobiological model of emotion regulation and sleep bruxism is highly innovative and has not been tested by others.
- Experimental manipulation of emotion regulation, including use of fMRI to identify the contribution of the amygdala and prefrontal cortex, in the context of sleep bruxism is also highly innovative.

Weaknesses

- The study design does not address the plausible alternate hypothesis that increased emotional dysregulation and stress (self-report, physiology) in SB+ patients might be a result, rather than cause, of the disorder.

4. Approach:

Strengths

- Clinical diagnostic criteria will be used to identify SB+ and SB- participants.
- A well-validated imaging task will probe the neurobiology of emotion regulation (ER) and differentiate ER from emotion generation. Imaging procedures (signal acquisition, processing, analyses) are similarly state-of-the-art.
- A well-defined multimodal protocol is proposed for the assessment of masticatory muscle activity (MMA) episodes and bruxism events during sleep.
- Preliminary data suggest that recruitment and enrollment are feasible. These data also support study hypotheses.
- Sex is considered as a biological variable, with approximately even numbers of men and women in the sample.
- Clear rationale for study measures and procedures will enhance the outcomes.
- Presentation of Critical Design Decisions addresses important issues of enhancing the rigor and reproducibility of study results, as well as associated limitations.

Weaknesses

- No major limitations noted.

5. Environment:

Strengths

- The research and infrastructure support at Stanford University are extremely well-suited to the conduct of the proposed randomized clinical trial.

Weaknesses

- No major limitations noted.

Protections for Human Subjects:

Acceptable Risks and/or Adequate Protections

- Clear and compelling presentation of plan.

Data and Safety Monitoring Plan (Applicable for Clinical Trials Only):

Not Applicable (No Clinical Trials)

Inclusion of Women, Minorities and Children:

- Sex/Gender: Distribution justified scientifically
- Race/Ethnicity: Distribution justified scientifically
- For NIH-Defined Phase III trials, Plans for valid design and analysis: Not applicable

Inclusion/Exclusion of Children under 18: Excluding ages <18; justified scientifically

Vertebrate Animals:

Not Applicable (No Vertebrate Animals)

Biohazards:

Not Applicable (No Biohazards)

Resource Sharing Plans:

Unacceptable

- Plan not included.

Authentication of Key Biological and/or Chemical Resources:

Not Applicable (No Relevant Resources)

Budget and Period of Support:

Recommend as Requested

CRITIQUE 3:

Significance: 2
Investigator(s): 1

Innovation: 1
Approach: 1
Environment: 1

Overall Impact: This is an exceptionally well written and cogently presented project to test a neurobiological model linking deficits in emotion regulation to sleep bruxism. The scientific premise is well articulated and compelling – although bruxism affects a small percentage of the general population, it can lead to a range of adverse outcomes – and the need for better understanding of its causes well articulated. The aims for the project are clear and proceed logically; indeed, the investigators effectively show that later aims do not rest critically on the success of earlier ones. Ample preliminary data are provided to support each of the aims, and the proposed experiments will provide rigorous tests of each hypothesis. The research team has the requisite expertise to accomplish the proposed work, resources necessary for the project are readily available at Stanford University, and the project seems both feasible and likely to have substantial conceptual and practical impact.

Protections for Human Subjects:

Acceptable Risks and/or Adequate Protections

Data and Safety Monitoring Plan (Applicable for Clinical Trials Only):

Not Applicable (No Clinical Trials)

Inclusion of Women, Minorities and Children:

- Sex/Gender: Distribution justified scientifically
- Race/Ethnicity: Distribution justified scientifically
- For NIH-Defined Phase III trials, Plans for valid design and analysis: Not applicable
- Inclusion/Exclusion of Children under 18: Excluding ages <18; justified scientifically

Vertebrate Animals:

Not Applicable (No Vertebrate Animals)

Biohazards:

Not Applicable (No Biohazards)

Authentication of Key Biological and/or Chemical Resources:

Not Applicable (No Relevant Resources)

Budget and Period of Support:

Recommend as Requested

THE FOLLOWING SECTIONS WERE PREPARED BY THE SCIENTIFIC REVIEW OFFICER TO SUMMARIZE THE OUTCOME OF DISCUSSIONS OF THE REVIEW COMMITTEE, OR REVIEWERS' WRITTEN CRITIQUES, ON THE FOLLOWING ISSUES:

PROTECTION OF HUMAN SUBJECTS (Resume): ACCEPTABLE
The protections plan was judged clear and appropriate.

INCLUSION OF WOMEN PLAN (Resume): ACCEPTABLE
The participants will comprise approximately equal numbers of women and men; this is scientifically justified.

INCLUSION OF MINORITIES PLAN (Resume): ACCEPTABLE
The plan was viewed as leading to a diverse sample, which was deemed a strength of the study.

INCLUSION OF CHILDREN PLAN (Resume): ACCEPTABLE
The study appropriately excludes children.

COMMITTEE BUDGET RECOMMENDATIONS: The budget was recommended as requested.

Footnotes for 1 R01 DE026771-01; PI Name: GROSS, JAMES J

NIH has modified its policy regarding the receipt of resubmissions (amended applications). See Guide Notice NOT-OD-14-074 at http://grants.nih.gov/grants/guide/notice-files/NOT-OD-14-074.html. The impact/priority score is calculated after discussion of an application by averaging the overall scores (1-9) given by all voting reviewers on the committee and multiplying by 10. The criterion scores are submitted prior to the meeting by the individual reviewers assigned to an application, and are not discussed specifically at the review meeting or calculated into the overall impact score. Some applications also receive a percentile ranking. For details on the review process, see http://grants.nih.gov/grants/peer_review_process.htm#scoring.
